# Structures, Phase Transitions and Tricritical Behavior of the Hybrid Perovskite Methyl Ammonium Lead Iodide

**DOI:** 10.1038/srep35685

**Published:** 2016-10-21

**Authors:** P. S. Whitfield, N. Herron, W. E. Guise, K. Page, Y. Q. Cheng, I. Milas, M. K. Crawford

**Affiliations:** 1 Chemical and Engineering Materials Division, Neutron Sciences Directorate, Oak Ridge National Laboratory, Oak Ridge, TN 37831, USA; 2 DuPont Electronics and Communication Technologies, Wilmington, DE 19803, USA; 3 DuPont Central Research & Development, Wilmington, DE 19803, USA; 4 Advanced Photon Source, Argonne National Laboratory, 9700 S. Cass Avenue, Lemont, IL 60439, USA; 5 Department of Physics and Astronomy, University of Delaware, Newark, DE 19716, USA

## Abstract

We have examined the crystal structures and structural phase transitions of the deuterated, partially deuterated and hydrogenous organic-inorganic hybrid perovskite methyl ammonium lead iodide (MAPbI_3_) using time-of-flight neutron and synchrotron X-ray powder diffraction. Near 330 K the high temperature cubic phases transformed to a body-centered tetragonal phase. The variation of the order parameter Q for this transition scaled with temperature T as Q ∼ (T_c_−T)^β^, where T_c_ is the critical temperature and the exponent β was close to ¼, as predicted for a tricritical phase transition. However, we also observed coexistence of the cubic and tetragonal phases over a range of temperature in all cases, demonstrating that the phase transition was in fact first-order, although still very close to tricritical. Upon cooling further, all the tetragonal phases transformed into a low temperature orthorhombic phase around 160 K, again via a first-order phase transition. Based upon these results, we discuss the impact of the structural phase transitions upon photovoltaic performance of MAPbI_3_ based solar cells.

The origin of the surprisingly high efficiencies exhibited by solar cells fabricated with hybrid perovskites remains a subject of widespread interest^1^,^2^. Photovoltaic (PV) efficiencies greater than 20% have been reported^3^, and these values were obtained using materials solution-processed near room temperature, which holds great promise for decreasing the cost of solar energy^4^.

To understand the origin of their PV performance, and accelerate the search for new materials, it is essential to first understand the crystal structures of the hybrid perovskites, characterized by structural phase transitions, considerable static or dynamic disorder, and unknown concentrations of various defects such as halogen anion or organic cation vacancies. The hybrid perovskites are far more complicated than conventional photovoltaic materials such as Si, CIGS, and CdTe, yet the perovskite structure is the basic framework adopted by a wide variety of functional materials such as ionic conductors, ferroelectrics and superconductors^5^. The parent perovskite structure ABX_3_ is cubic with *Pm-3m* symmetry but may lower its symmetry by rotating or distorting the ‘BX_6_’ octahedra and translating the ‘A’ site or ‘B’ site cations. The structures obtained by simple rotations of the BX_6_ octahedra around the axes of the aristotype cubic structure were first classified by Glazer^6^, followed by a number of theoretical studies of the possible tilt structures and phase transitions^7^,^8^,^9^,^10^.

The hybrid perovskite MAPbI_3_ (CH_3_NH_3_PbI_3_) was first synthesized and described by Weber in 1978^11^ as an analog of CsPbI_3_^12^. MAPbI_3_ was shown^13^ to have three structural phases: a cubic *Pm-3m* phase above 330 K, a tetragonal *I4/mcm* phase from 160 to 330 K, and an orthorhombic *Pnma* phase below 160 K. The structural phase transitions connecting these phases can be rationalized within the schemes used to describe transitions in perovskites with inorganic cations^6^,^7^,^8^,^9^, but for MAPbI_3_ order-disorder transitions of the MA cations are also involved.

To improve our understanding of the crystal structures and phase transitions in MAPbI_3_, we performed detailed structural studies using both time-of-flight neutron and synchrotron X-ray powder diffraction. Although there have been several studies of MAPbI_3_ using reactor-based neutron powder diffraction^14^,^15^, those studies did not utilize deuterated samples to eliminate the strong incoherent scattering from hydrogen, nor did they fully address the structural complexity introduced by the MA cations in this perovskite. To our knowledge, there also have not been any temperature-dependent studies of MAPbI_3_ that took advantage of the higher resolution of synchrotron X-ray powder diffraction to study the structural phase transitions in detail. The current studies showed some surprising features having implications for the use of MAPbI_3_ as a PV absorber.

## Results

### Structure of the Orthorhombic Phase: Fully Ordered MA Cations

**Figure 1:**
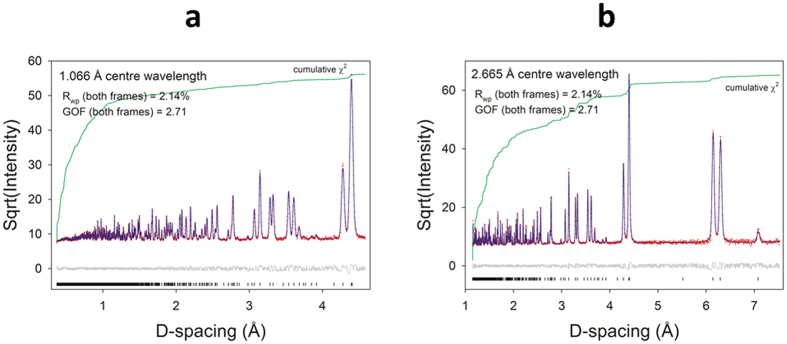
Neutron powder diffraction data and structure refinement for d_6_-CD_3_ND_3_PbI_3_ at T = 10 K. Data shown were collected using (**a**) 1.066 Å and (**b**) 2.665 Å wavelength neutrons. The data points are shown in red, the Rietveld fit in blue, the residual in gray, and the cumulative χ^2^ in green. The black vertical tick marks indicate the expected Bragg peak positions. A small amount of residual diffuse background is visible in the difference curve at low d-spacing.

Figure 1 shows the Rietveld refinements of the neutron powder diffraction (NPD) data measured at 10 K. The structure was found to be similar to d_3_-CH_3_ND_3_PbBr_3_ at 11 K^16^ and h_6_-MAPbI_3_ at 100 K^14^, but the lower temperature resulted in less thermal motion than observed in the 100 K data^16^. The use of a fully deuterated sample yielded data with superior signal-to-background, and data collection to higher Q improved the accuracy and precision of structural parameters. Some diffuse scattering was visible in the background even at 10 K but a fully un-constrained refinement with anisotropic displacement parameters (ADPs) could still be carried out. The refined structural parameters are shown in Supplementary Table 1. Density Functional Theory (DFT) was used to verify the cell symmetry by optimizing the refined 10 K structure in *P1* symmetry. The calculation converged rapidly, indicating the refined structure was extremely close to the energy minimum. Optimized lattice parameters from VASP are compared to the refined values in Supplementary Table 2. The errors were less than 1%.

Data were also obtained at 10 K from partially deuterated d_3_-CD_3_NH_3_PbI_3_ and d_3_-CH_3_ND_3_PbI_3_ to determine whether deuteration had a significant effect on the structures or phase transitions. Increased background from the incoherent scattering of hydrogen was present (Supplementary Figs 1 and 2) but full, unconstrained Rietveld refinements including ADPs could still be carried out. The structures were essentially identical to that of d_6_-MAPbI_3_. As expected, thermal ellipsoids for hydrogen were slightly larger than for the heavier deuterium atoms (Supplementary Figs 1 and 2). Various bond lengths and angles for the three samples at 10 K are shown in Supplementary Table 3. There were subtle differences between the structures. As expected, bonds between ‘H’ and I were slightly longer with hydrogen versus deuterium but still comfortably within the range of values observed for N(sp^3^)-H-I hydrogen bonds^17^. Although there were significant differences in bond lengths and angles making up the N-(D/H) I hydrogen bonds, there were no differences in the overall N-I(1) distances. The most striking difference was the I(1)-Pb-I(2) bond angle, which showed how motion of the apical iodine distorted the PbI_6_ octahedra. When bonded to hydrogen the I(1)-Pb-I(2) bond angle distorted by nearly 3 degrees more than when bonded to deuterium. The C-N bond length of 1.499(2) Å in the fully deuterated phase closely matches the 1.497(2) Å C-N bond length in d_6_-ND_3_CD_3_GeCl_3_^18^. The shorter C-N bonds of the partially deuterated phases also match those for partially deuterated d_3_-MAPbBr_3_^16^.

**Figure 2:**
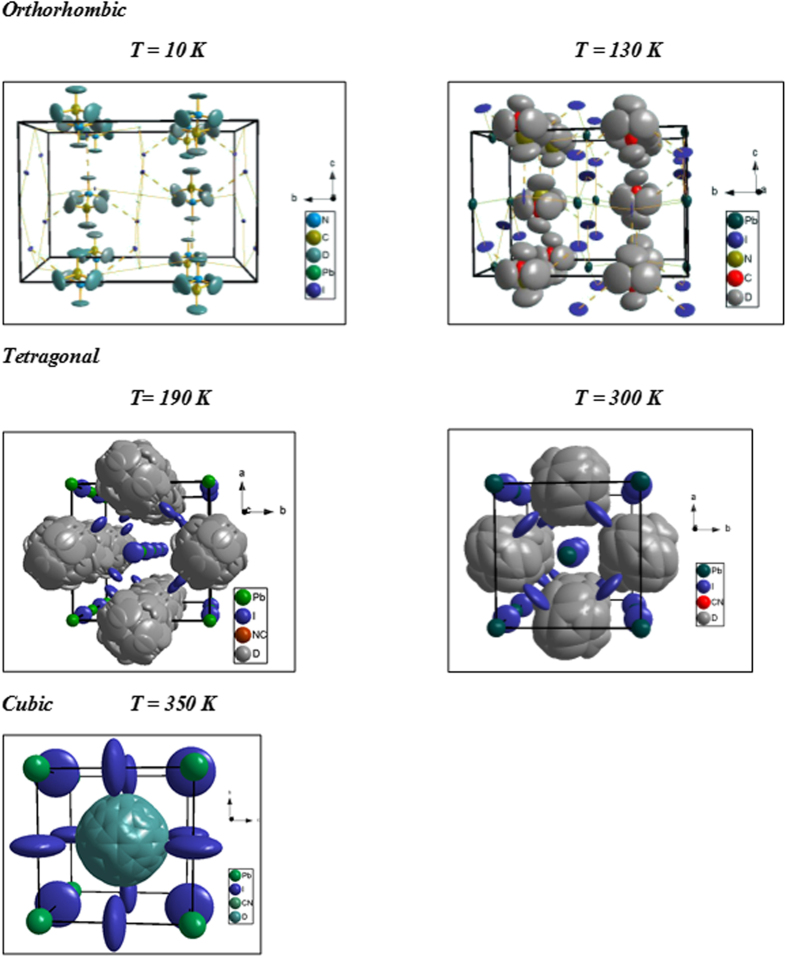
Structures of d_6_-MAPbI_3_ at five temperatures based upon refinement of neutron powder diffraction data. Ellipsoids are drawn at 95% probability. The increase of MA disorder with temperature is evident, as is the increasing size of the iodine atom ADPs due to rotational disorder of the PbI_6_ octahedra.

The lower signal-to-noise ratio and increased diffuse scattering of the 130 K data for d_6_-MAPbI_3_ (Supplementary Fig. 3) meant TLS restraints^19^ were required to obtain stable ADPs from the MA cation. The structure was very similar to the 10 K structure except for the enlarged ADPs. The iodine atoms began to show the anisotropic motion seen in the tetragonal and cubic phases, as seen in Fig. 2.

### The Structure of the Tetragonal Phase: Partially Ordered MA Cations

**Figure 3:**
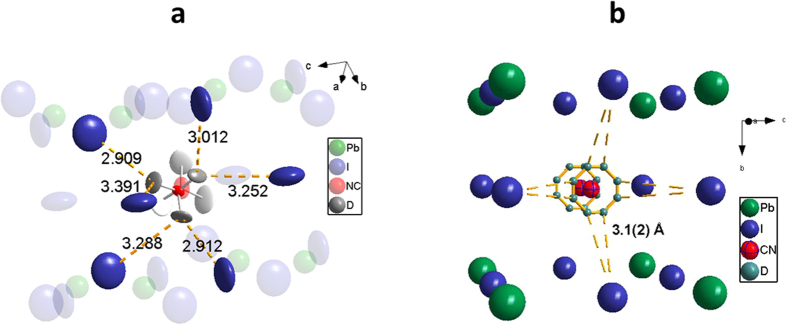
Illustration of the orientations of the MA cations in the tetragonal and cubic structures. (**a**) Projection along the C-N bond of the d_6_-MA cation for the 190 K *I4/mcm* structure, showing its bonding environment at the less-mobile end of the MA cation. The thermal ellipsoids for the MA cation are 25% probability and the rest of the image at 95% probability. At first glance the nearest coordination lengths at the more mobile end of the MA cation are noticeably longer with an average of 3.532 Å compared to 3.127 Å for the less mobile end. However, the end-to-end disorder means that on average the real coordination lengths are the same in all directions. (**b**) A single orientation of the MA cation is shown for the cubic phase at 350 K.

In common with previous studies^14^,^15^, the space-group *I4/mcm* was used for the tetragonal phase. Since the MA cation has C_3v_ symmetry it will be disordered on the Wyckoff 4(b) site at (½, 0, ¼), having 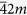 (D_2d_) point group symmetry (the point midway between the C and N atoms at T = 190 K is located slightly off the 4(b) site at (0.5265, −0.0265, 0.2406) or symmetry related positions). Quasielastic neutron scattering measurements clearly showed the disorder to be dynamic^20^,^21^. For this reason, together with fewer usable Bragg reflections, refinement of the tetragonal structure was more difficult than refinement of the fully ordered orthorhombic structure. For example, the MA cation could be disordered end-to-end, distributed over eight orientations in the unit cell, or ordered end-to-end, leading to only four orientations. The four-fold model was used for previous refinements in *I4/mcm* symmetry^14^,^22^, but heat capacity^23^ and NMR^24^,^25^ studies suggested MA has eight-fold disorder in the tetragonal phase, at least at the low end of the temperature range of tetragonal stability. A recent report based upon neutron single crystal data proposed an alternative disorder for the MA where nitrogen remained ordered on the mirror plane and the methyl group disordered off the mirror plane^26^. Differentiating unambiguously between 4-fold and 8-fold disorder of the MA cation wasn’t possible with our diffraction data alone. The weighted-profile R values (R_wp_) for the four-fold and end-to-end eight-fold models were 2.42% and 2.34%, respectively, the latter model assuming 50:50 disordered N and C. Refining the C:N disorder in the tetragonal data suggested some temperature dependence of the C:N ratio on each site, but the refinement was not stable. Applying instead the structural model from ref. 26 to the tetragonal data produced good fits but we encountered issues with inconsistent bond lengths and ADPs that prevented a definitive conclusion as to which eight-fold model is best. For consistency with thermodynamic and NMR data, the simple end-to-end eight-fold disorder model was adopted (with C and N both on the mirror plane), together with the constraint that the C/N to D bond lengths were equal at both ends of the molecule. Given that they are not expected to be equal, this assumption will affect the ADPs of the deuterium atoms. The asymmetric motion of MA in Fig. 3a suggests complexity beyond this simple 50:50 C:N disorder model.

The refinement fit is shown in Supplementary Fig. 4 and the structure in Fig. 2. The eight-fold disorder makes it difficult to visualize the MA environment, so Fig. 3a displays a single molecular orientation. The MA adopted a staggered conformation in the perovskite, as expected for an ethane-like molecule. The dihedral angle of the MA cation was 186.9° rather than the 180° of the ideal staggered conformation. H-bonds are known to affect torsion angles, the magnitude dependent upon the torsional energy barrier and H-bond strength^17^. The magnitude of the effect in this case suggests a significant H-bond interaction despite the relatively long D-I(2) distance of 2.909(5) Å. It should be noted that in ref. 14, the MA conformation in the tetragonal phase is eclipsed, in disagreement with the results reported here. The final refined atomic coordinates are shown in Supplementary Table 4. The refined C-N bond length was 1.493(4) Å, consistent with the 1.499(2) Å found at 10 K.

The fit to the 300 K data for the tetragonal structure is seen in Supplementary Fig. 5. The diffuse scattering at high Q was more significant, and considerable background misfit remained even after applying a sin(Qr)/Qr background, implying that a single correlation length did not fully characterize the diffuse scattering. To avoid over-parameterization, the Rae^27^ TLS parameters relating to screw motion were set to zero. This stabilized the refinement, yielding ADPs with the expected magnitudes versus the 190 K refinement. Obviously fixing the screw-terms to zero removed the twisting observed in Fig. 3a, but refinement of the TLS center-of-action still resulted in asymmetric motion of the MA.

### The Structure of the Cubic Phase: Fully Disordered MA Cations

The cubic phase of MAPbI_3_ has *Pm-3m* space group symmetry. As the MA cation has C_3v_ symmetry, it is not possible to place it on Wyckoff site 1(b) at (½, ½, ½) with octahedral m-3m (O_h_) point group symmetry without inducing disorder. In cubic MAPbI_3_ the MA cations disorder over six equivalent orientations, with the C-N bond axis aligned along any of the <100> directions. One of these orientations is illustrated in Fig. 3b. The presence of the MA three-fold axis is inconsistent with the four-fold symmetry axes required for this octahedral site, so MA must exhibit four-fold rotational disorder around these axes. This led to 24 possible molecular orientations of the MA cation similar to that reported by Weller^14^ for CH_3_NH_3_PbI_3_. Thus the number of equivalent orientations determined from our neutron diffraction data for the cubic (24), tetragonal (8) and orthorhombic (1) structures were fully consistent with changes in entropy extracted from heat capacity data^23^. The suggestion that the high temperature phase is tetragonal with *P4mm* symmetry^15^,^28^ would not be consistent with thermodynamic measurements, nor did we find any improvement in the quality of Le Bail fits in *P4mm* versus *Pm-3m* symmetry.

The cubic *Pm-3m* structure had a minimal number of structural variables compared to the tetragonal phase (Supplementary Table 4). The structure (Fig. 2 and Supplementary Fig. 6) had a freely-rotating MA cation, yielding very large refined displacement parameters (Supplementary Table 5). The distances between deuterium and iodine atoms of 3.1(2) Å shown in Fig. 3b suggest the disordered MA cations still had a weak hydrogen bonding interaction with the PbI_6_ framework in the cubic structure.

### Local Structure From Pair Distribution Function (PDF) Analysis

**Figure 4:**
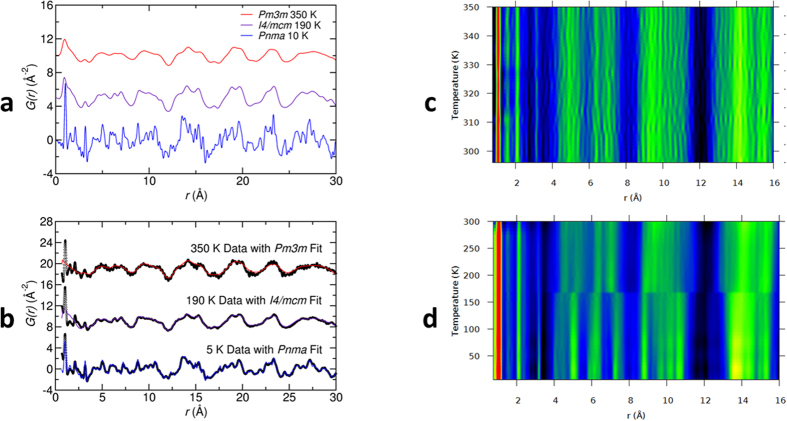
Pair correlation functions G(r) for three crystal structures of d_6_-MAPbI_3_. (**a**) Simulated G(r) for the orthorhombic, tetragonal and cubic phases calculated from the 10, 190 and 350 K refined atomic coordinates from POWGEN data. (**b**) G(r) from pair distribution function analysis of NOMAD data for cubic, tetragonal and orthorhombic phases. Neutron PDF contour plots showing: (**c**) minimal changes in the local atomic structure (0.75 Å to 16 Å) across the tetragonal-to-cubic phase transition on warming from 296 K to 350 K in ~5 K steps; and (**d**) minimal changes in the first 4 Å of the local atomic structure across the entire 5 K to 300 K temperature range on warning in ~25 K steps, with strong changes in the 4 Å and above local atomic structure at the orthorhombic to tetragonal phase transition at approximately 170 K. Differences in data noise level in (**c,d**) reflect differences in sample size, measurement time, and sample environments for the two series.

Very few local structure studies of MAPbI_3_ have been reported: bulk and nanocrystalline structures have been studied via X-ray PDF studies at room temperature utilizing data above 3 Å, excluding H atoms (due to X-ray insensitivity), and using tetragonal space group *I4cm*^29^. Neutron PDF analysis over a broad temperature range allowed us to examine the local atomic structure in more detail. The calculated G(r) from the refined average structures (simulated utilizing the 10 K, 190 K, and 350 K structures reported in Supplementary Tables 1,4 and 5) are shown in Fig. 4a and the fits to the experimental G(r) for the orthorhombic, tetragonal and cubic phases of d_6_-MAPbI_3_ at 5 K, 190 K and 350 K are shown in Fig. 4b. The calculated G(r)s are distinct across the full range of PDF distances, displaying significant thermal broadening of pair-pair correlations at higher temperatures, while the experimental patterns were almost identical up to approximately 3 Å. This behavior is more clearly displayed in Fig. 4c,d where the G(r) is shown for a 2-D mapping of the data series. There was no obvious transition in the pair distances observed over the tetragonal-to-cubic transition in Fig. 4c; rather, only gradual thermal broadening is observed. However, in Fig. 4d the orthorhombic-to-tetragonal transition at ~160 K is indicated by an abrupt break in the data. It is striking that minimal changes were observed across the entire temperature range in the region below 4 Å where the intramolecular atom-atom distances for the MA cation are located. At 10 K the shortest Pb-I distance was just over 3 Å, and the shortest D-I bond observed from the structure refinements was 2.6–2.7 Å. Local contributions to the atomic structure fits to the 10 K data shown in Fig. 4b are provided in Supplementary Fig. 7. The detailed local structure behavior of MAPbI_3_ is complex and modeling of the complete data series will be the subject of a future contribution.

### Structural Phase Transitions: Distortion Mode Analysis and Order Parameters

**Figure 5:**
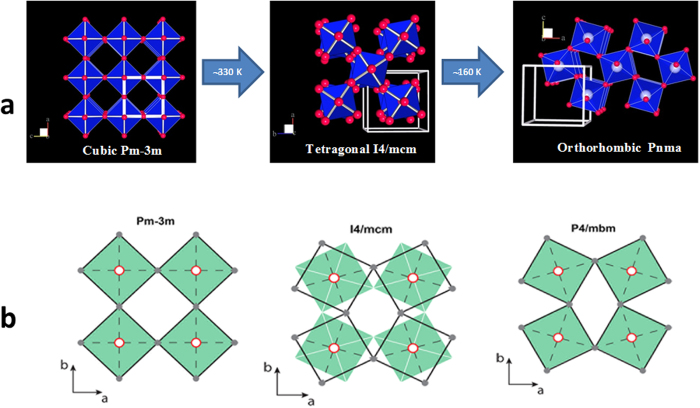
The crystal structures adopted by MAPbI_3_. (**a**) The PbI_6_ octahedra are blue and the iodine atoms are red. The cubic to tetragonal transition is due to the R_4_^+^ rotational distortion mode, and the tetragonal to orthorhombic transition is primarily associated with a combination of the R_4_^+^ and M_3_^+^ distortion modes. In this figure the MA cations are not shown to better highlight the distortions of the Pb-I network due to the structural phase transitions. (**b**) The relative rotations of neighboring layers of PbI_6_ octahedra along the *c* axis are shown as filled green squares and unfilled black squares. In the cubic *Pm-3m* structure the octahedra are not rotated. In the *I4/mcm* structure the octahedra in neighboring planes along the *c* axis rotate in the *opposite* sense, whereas in the *P4/mbm* structure the neighboring planes of octahedra along the *c* axis rotate in the *same* sense.

The structural phase transitions in MAPbI_3_ are similar to other perovskites such as SrTiO_3_^30^ and KMnF_3_^31^; yet complicated by the presence of low symmetry (C_3v_) organic cations and their order-disorder transitions coupled with tilts of the PbI_6_ octahedra. Despite this, the essential features of the phase transitions in MAPbI_3_ may still be described using the formalism developed for inorganic perovskites^6^,^7^,^8^,^9^,^10^. The dominant component of these phase transitions is rigid rotation of the PbI_6_ octahedra. Since the octahedra share corners, rotations must be out-of-phase in directions ***perpendicular to*** this rotation axis, but may be in-phase or out-of-phase ***parallel to*** this axis (Fig. 5). If rotations are in-phase along this axis, the unit cell parameter along this axis does not change; if rotations are out-of-phase, the unit cell parameter doubles. These pure tilt transitions are located at the Brillouin zone (BZ) boundaries of the cubic unit cell: the in-phase rotations are at the M-point = (½ ½ 0) and the out-of-phase rotations are at the R-point = (½ ½ ½). When the octahedra undergo rotations that are not perfectly in-phase or out-of-phase, the transitions occur along the line T connecting the M and R points of the BZ. Since these phase transitions lead to new lattice vectors and larger unit cells, the M or R points fold over to the Γ point in the lower symmetry phase (which has *P4/mbm* or *I4/mcm* symmetry for the M-point or R-point instability, respectively). This information is summarized in Table 1.

In the lower temperature phases new superlattice Bragg peaks appear indicating the type of distortion^6^,^7^,^9^. The R-point transitions lead to superlattice peaks indexed to the doubled pseudocubic cell as (*h k l*), with *h*, *k* and *l* all odd; the M-point superlattice peaks instead have one even and two odd. The integrated intensities of the superlattice peaks (I_SL_) are expected to scale as the square of the order parameter for these phase transitions, I_SL_ ∼ Q^2^ ∼ (T_c_ – T)^2β^, where the order parameter is the rotation angle around a particular axis of the PbI_6_ octahedra referenced to the aristotype structure. Secondary order parameters such as the tetragonal strain in the *I4/mcm* structure will also scale as the square of the order parameter, (*c−a*)/*a* ∼ Q^2^ ∼ (T_c_ – T)^2β^ 32,33. Thus our diffraction data can be used not only to determine the crystal structures of the different phases, but to measure the order parameters for the phase transitions. These values can then be compared to predictions from models for critical behavior^33^ to understand the nature of each phase transition.

A related approach is to use distortion mode analysis^34^ to decompose structural changes that carry the aristotype *Pm-3m* structure to lower temperature, less symmetric hettotype structures (*I4/mcm* and *Pnma*) in terms of symmetry adapted basis vectors (normal modes) of the cubic structure. This procedure provides a group theoretical understanding of the phase transitions, as well as insight into the specific phonon branches participating in the phase transitions^35^. This approach may be combined with a multi-dataset surface refinement where distortion mode amplitudes are extracted while approaching the phase transition.

Despite the good resolution provided by POWGEN, the increased resolution offered by synchrotron X-ray powder diffraction allowed a more detailed examination of phase compositions across the phase transitions, and also provided the temperature dependence of the order parameter associated with each transition. Although not very sensitive to the positions of the organic cations, X-rays are very sensitive to the heavier PbI_6_ octahedra and their rotations

### Cubic-Tetragonal Phase Transition

**Figure 6:**
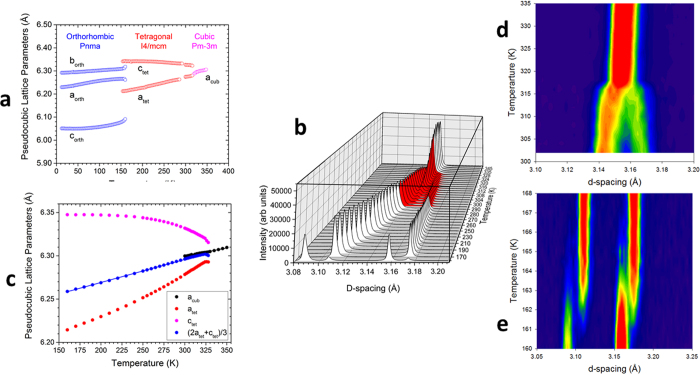
Lattice parameters and phase transitions of d_6_-MAPbI_3_. (**a**) Lattice parameters refined from datasets sliced from two continuous POWGEN datasets collected using a ramp-rate of 1 K/min, the first between 10–300 K in the sample changer and the second from 300–350 K in the Janis cryofurnace. (**b**) Bragg peaks across the cubic-tetragonal phase transition measured by synchrotron X-ray powder diffraction. The (200) cubic Bragg peak and the (220)/(004) tetragonal Bragg peaks are shown as a function of temperature. There is a wide range of cubic and tetragonal phase coexistence from 300–330 K illustrated by the peaks colored red. At 160 K the sample is mostly *Pnma* with a small amount of *I4/mcm* still present. (**c**) Lattice parameters determined from the synchrotron X-ray diffraction scans of the cubic 200 and tetragonal 220/004 Bragg peaks. Fits to the cubic lattice parameter (black solid line) and the average tetragonal lattice parameter (=2a_tet_ + c_tet_)/3 (blue solid line), were used to determine the linear coefficients of thermal expansion of 1.95 × 10^–4^ K^−1^ (cubic) and 2.66 x 10^−4^ K^−1^ (tetragonal). The region of cubic and tetragonal phase coexistence can be clearly seen. (**d**) Neutron diffraction contour plot showing the tetragonal 220 and 004 reflections approaching the tetragonal-to-cubic phase transition on cooling at 0.5 K/min. The data was binned every 0.5 K. (**e**) Neutron diffraction contour plot showing the region of the orthorhombic 202 and 040 reflections approaching the phase transition on cooling at 0.25 K/min. The contour scale has been chosen to better show the weak intensities.

Upon cooling the cubic phase, MAPbI_3_ undergoes a transition to tetragonal symmetry near 330 K (Figs 3 and 6). This phase transition, from *Pm-3m* to *I4/mcm* symmetry, can be second-order within Landau theory since the space group *I4/mcm* is a subgroup of *Pm-3m*^36^. Several studies have addressed the order of this phase transition but reached different conclusions: it was first-order based upon specific heat data^23^; second-order based upon neutron diffraction^14^; or possibly close to tricritical based upon single crystal X-ray diffraction^22^. Tricritical points separate lines of first-order and second-order phase transitions and are characterized by a critical exponent β of ¼ 33 (also see Supplementary Information). We closely examined this phase transition using time-of-flight neutron powder diffraction and high resolution synchrotron X-ray powder diffraction in order to clarify its nature.

**Figure 7:**
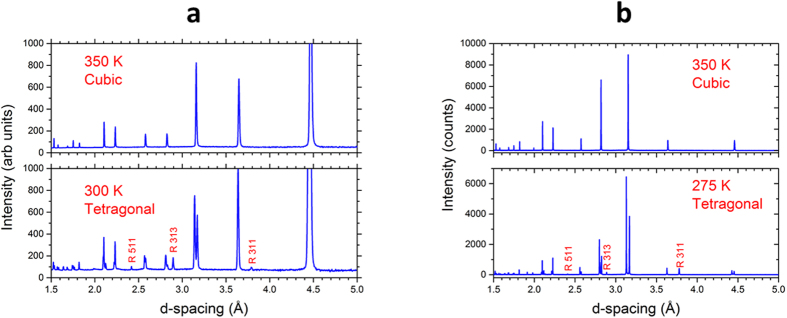
Neutron and x-ray diffraction patterns of d_6_-MAPbI_3_ illustrating the R-point superlattice Bragg reflections that appear due to the transition from the cubic *Pm-3m* to the tetragonal *I4/mcm* structure. (**a**) Neutron powder diffraction patterns of the cubic and tetragonal phases of d_6_-MAPbI_3_ showing the locations of several R-point superlattice peaks (labeled) that appear for the tetragonal phase as a result of the out-of-phase rotations of the PbI_6_ octahedra. (**b**) Synchrotron X-ray powder diffraction patterns of d_6_-MAPbI_3_ showing R-point superlattice peaks. The peaks are indexed in the cubic cell with doubled lattice parameters.

Figure 7 shows neutron and X-ray diffraction patterns of the cubic and tetragonal phases of d_6_-MAPbI_3_. Peak-splitting due to the tetragonal distortion (strain) and new superlattice peaks could be seen in both sets of data. All of the observed superlattice peaks could be indexed in the doubled pseudocubic cell as (*h k l*), with all three indices odd. Thus only out-of-phase PbI_6_ rotations were present (Fig. 5), due to condensation of a single triply degenerate R_4_^+^ phonon mode at the R point of the cubic Brillouin zone. All peaks were readily indexed in tetragonal symmetry, with an enlarged unit cell having the pseudocubic lattice parameters given in Table 1.

**Figure 8:**
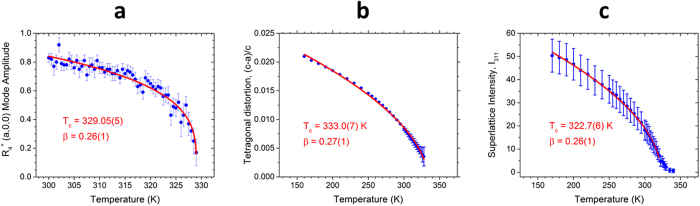
Temperature dependence of the order parameter (out-of-phase rotation angle for the PbI_6_ octahedra about the cubic *Pm-3m a* axis) for the cubic-tetragonal phase transition of d_6_-MAPbI_3_. (**a**) The R_4_^+^ rotational mode amplitude, determined by distortion mode analysis of the neutron diffraction data, which is directly proportional to the cubic-tetragonal order parameter, is fit to a power law, R_4_^+^ ∼ (T_c_−T)^β^, where β is the critical exponent. (**b**) Tetragonal distortion from X-ray powder diffraction data fit to a power law (red line) for a secondary order parameter that is coupled to the primary order parameter: (c−a)/a ∼ (T_c_−T)^2β^. Note the very different temperature ranges over which the data in (**a,b**) were collected. Data collected while cooling. Error bars were multiplied by 3 for better visibility. (**c**) Temperature dependent intensity of the R-point (311) superlattice Bragg reflection measured using synchrotron X-ray powder diffraction. The intensity of this peak will be affected by the magnitude of the out-of-phase rotation and by the amount of tetragonal phase present. At temperatures below 300 K the sample is almost completely tetragonal (no cubic phase remains according to the X-ray data). The red line is a fit to a power law, I_311_ ∼ (T_c_−T)^2β^. Data collected while cooling.

In order to examine the cubic-tetragonal transition more closely, symmetry-mode decomposition was carried out in ISODISTORT^34^ for the 190 K tetragonal PbI_3_ framework versus the 350 K cubic framework. The temperature dependence of the order parameter, i.e. rotation angle of the PbI_6_ octahedra (equivalent to the R_4_^+^ distortion mode amplitude), was determined by binning the continuous neutron diffraction dataset every 0.5 K up to the phase transition and performing a surface refinement to extract the R_4_^+^ mode amplitude as a function of temperature. The temperature dependence of R_4_^+^ is seen in Fig. 8. The data were fitted to a power–law, R_4_^+^ ∼ (T-T_c_)^β^, to determine the critical temperature T_c_ = 329.05(5) K and exponent β = 0.26(1). This value of β is very close to that expected for a tricritical point^33^, and is also close to the value of 0.22(3) determined by single crystal X-ray diffraction for hydrogenated h_6_-MAPbI_3_^22^. Together with data for polycrystalline samples described below these β values suggest that the near-tricritical behavior is independent of hydrogen isotope.

The tetragonal distortion was analyzed using synchrotron X-ray powder diffraction for fully deuterated d_6_-MAPbI_3_ (and fully hydrogenated h_6_-MAPbI_3_) by measuring (1) the splitting of the 220 and 004 Bragg reflections derived from the cubic 200 reflection, shown in Fig. 6b for d_6_-MAPbI_3_ (and in Supplementary Fig. 8a for h_6_-MAPbI_3_); and (2) measuring the intensity of the R-point (311) superlattice Bragg reflection (referenced to the cubic unit cell doubled along *a*, *b* and *c* axes; this would be the 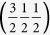 Bragg reflection in the standard cubic cell), shown in Supplementary Fig. 9 for d_6_-MAPbI_3_ (and in Supplementary Fig. 8b for h_6_-MAPbI_3_). Both measurements came to a similar conclusion, as shown in Fig. 8 for d_6_-MAPbI_3_ and in Supplementary Fig. 10 for h_6_-MAPbI_3_. The cubic-tetragonal transition was in all cases close to tricritical, independent of hydrogen isotope composition. However*, the X-ray data for d*_*6*_*-MAPbI*_*3*_ (Fig. 6b) *showed very clearly that the cubic and tetragonal phases coexisted over a wide temperature range (nearly 30 K), proving that this phase transition was first-order*. We also verified that the transition in h_6_-MAPbI_3_ was first-order, as shown in Supplementary Fig. 8a, with a slightly narrower temperature range (ΔT~15 K) of cubic/tetragonal phase coexistence. This observation has important implications for use of MAPbI_3_ as a PV absorber since the temperature range of phase coexistence is well within the temperature range for PV applications.

### Tetragonal-Orthorhombic Phase Transition

Decomposition of the refined 10 K structure with ISODISTORT^34^ showed the major displacement modes in the orthorhombic structure to be the pure octahedra tilts R_4_^+^ and M_3_^+^ described previously. In addition to R_4_^+^ and M_3_^+^ described in Table 1, the *Pnma* structure can include three other distortion modes: R_5_^+^, X_5_^+^ and M_2_^+^ 10. However, these modes made minimal contributions to the orthorhombic structure so are described in the Supplementary Information.

The orthorhombic-to-tetragonal phase transition was first-order from *Pnma* to *I4/mcm* symmetry, with co-existing phases as seen in the lattice parameter plot and contour plots shown in Fig. 6. The fact that *Pnma* is not a subgroup of *I4/mcm* means a continuous second-order phase transition is not possible. The *Pnma* to *I4/mcm* transformation requires modes with two different irreducible representations to condense (Table 1) and thus has two primary order parameters, violating one of the Landau conditions for a second-order phase transition^37^.

The refined tetragonal and orthorhombic phase fractions versus temperature are shown in Supplementary Fig. 11. In order to model the behavior observed in the data the phase fractions from 169–175.5 K were parameterized using the Kolmogorov-Johnson-Mehl-Avrami rate equation (see Methods). The data could be fitted using an exponent of 2.5 and a refined rate constant of 0.0014 s^−1^. An Avrami exponent (m+1) of 2.5 corresponds to a diffusion-controlled, homogeneous nucleation process^38^. The slope of a plot of ln[-ln(1-wt%_tet_)] vs 1/T for the results between 169 and 171 K yielded an activation energy of 23.5 kJ/mol for the transition, typical for solid-solid transitions involving molecular reorientation^39^.

## Discussion

Neutron powder diffraction and high resolution synchrotron X-ray diffraction measurements of fully deuterated MAPbI_3_ were performed. Refined structures, including ADPs, were obtained for the cubic, tetragonal and orthorhombic phases from neutron diffraction data. The combination of a fully deuterated sample and high Q-range accessible with time-of-flight neutron data produced very high quality structural information. Additionally, orthorhombic structures at 10 K for the partially deuterated phases d_3_-CD_3_NH_3_PbI_3_ and d_3_-CH_3_ND_3_PbI_3_ were refined and found to be essentially identical to that of d_6_-MAPbI_3_. With respect to possible ferroelectric behavior in MAPbI_3_, reducing the symmetry from centrosymmetric *Pm-3m* to polar *P4mm*^28^ did not improve the refinement in any meaningful way, which rules out the possibility of long-range ferroelectric behavior.

Based upon results of high-resolution synchrotron X-ray powder diffraction measurements, the cubic-tetragonal phase transition was shown to be first-order, with a range of temperature over which both phases coexisted for fully deuterated or fully hydrogenated samples. However, both neutron and X-ray data revealed a transition whose order parameter behaved close to that predicted for a tricritical phase transition. Tricritical behavior for the *Pm-3m* → *I4/mcm* transition has been seen in many other perovskites, for example SrZrO_3_^40^, and can be accounted for by a Landau potential energy function including terms to sixth-order in the order parameter^33^, as described in the Supplementary Information. Tricritical behavior occurs when the fourth-order term vanishes, leading to a critical exponent β = ¼. However, the same Landau theory also predicts a vanishing region of phase coexistence at the tricritical point^37^, which clearly disagrees with our observations. Possible reasons for this are the importance of higher order terms in the Landau expansion^37^, or the effect of critical fluctuations on the cubic-tetragonal phase transition^41^. Critical fluctuations of the triply-degenerate R_4_^+^ zone-boundary mode lead to first-order behavior for the *Pm-3m* → *I4/mcm* transition in a number of inorganic perovskites^41^. In a different scenario, effective coupling between the MA cation order and octahedral tilt instabilities can also influence the behavior in a way that is not properly accounted for by a simple 2-4-6 Landau potential appropriate for inorganic materials^42^. Additional measurements, including measurements of phonon dispersion curves and evaluation of the possible influence of defects^33^, will further clarify the contributions to its complex structural phase transitions.

The low temperature tetragonal-to-orthorhombic phase transition was also first-order, but exhibited phase coexistence over a narrower temperature range. Temperature-dependence of the mode amplitudes during the orthorhombic-to-tetragonal transition exhibited possible differences in transition temperatures for some modes, but were on the edge of statistical significance.

Both transitions exhibited thermal hysteresis that varied with heating or cooling rate, as expected for first-order phase transitions (Table 2). In addition, there were apparent isotope effects on the phase transition temperatures, and on the temperature range of the phase coexistence region for the cubic-to-tetragonal phase transition (Table 2). However, the isotope effects were quite small, typically less than 10 K for MAPbI_3_ after complete deuteration (Table 2). Although similar phase transitions occur in other inorganic perovskites^40^, it is interesting to note that in formamidinium (CH(NH)_2_) lead iodide (FAPbI_3_), the high temperature cubic structure transforms to a different tetragonal structure than MAPbI_3_^28^, with *P4/mbm* space group symmetry^43^. This transition, driven by condensation of an M-point soft mode (Fig. 5b), is similar to the high temperature transition observed in CsPbCl_3_^44^. The choice of organic cation clearly influences the relative stabilities of different tilt structures in these materials.

Our PDF data showed little change in local structure across the cubic-tetragonal phase transition. This suggests the cubic phase is locally similar to the tetragonal phase up to 350 K, consistent with the observed minimal changes in the electronic or dielectric properties over this temperature range. It is likely that this tetragonal distortion is dynamic in nature, reflecting fluctuations due to the R-point soft modes in the cubic phase. With decreasing temperature the soft mode driven tetragonal fluctuations grow larger, slow down and then freeze, leading to the appearance of the average tetragonal structure shown by conventional diffraction measurements. Thus the cubic-tetragonal transition appears to be primarily displacive in character despite the possible coupling to the MA cation order. Whether there is also an order-disorder component of this phase transition associated with the octahedral tilts is a question that requires more careful modeling of the PDF data, but we note that the *Pm-3m* to *I4/mcm* transition has been described as an order-disorder transition in several inorganic perovskites^45^,^46^.

In contrast to the cubic-tetragonal transition, the tetragonal-orthorhombic transition in MAPbI_3_ is primarily an order-disorder transition of the MA cations coupled strongly to the Pb-I framework through hydrogen bonds, leading to the orthorhombic distortion of the tetragonal lattice. This transition appears in both the local and long-range structures and has a large impact on the electronic properties of MAPbI_3_.

Do the structural phase transitions of MAPbI_3_ affect PV performance? A number of studies have investigated the impact of the phase transitions on optical^47^,^48^,^49^, thermal^23^, dielectric^50^, and PV^51^ properties. In addition, the electronic structures of MAPbI_3_ have been calculated for the cubic, tetragonal, and orthorhombic structures^52^. In general, experiments show relatively minor effects at the cubic-tetragonal transition, but considerably larger effects at the tetragonal-orthorhombic transition. It is clear that the electronic properties are primarily associated with the Pb-I framework, and changes in those bond lengths and angles will impact the PV properties. The cubic-tetragonal phase transitions in our samples are of first-order, with a significant region of phase coexistence. Within the coexistence region the samples are composed of regions of average cubic and tetragonal symmetries, as shown by the presence of sharp Bragg peaks for both phases, that become fully tetragonal (cubic) at lower (higher) temperatures. Although the average structure changes at the cubic-tetragonal phase transition, our PDF results show that the local structure does not change significantly, suggesting that the Pb-I bond distances and angles are not greatly affected, consistent with the reported insensitivity of various electronic properties to this phase transition. However, there are other implications of the phase transition that are important to consider.

In the tetragonal phase there will be three different tetragonal domains, according to which cubic axis in *Pm-3m* symmetry has become the unique tetragonal c axis in *I4/mcm* symmetry. These three tetragonal domains are separated by domain walls, where the axis about which the PbI_6_ octahedra rotate changes from that characteristic of one domain to that characteristic of another, each domain the result of the condensation of a different (x, y or z) component of the three-fold degenerate R_4_^+^ mode located at the R-point of the Brillouin zone This zone boundary phonon mode involves out-of-phase rotations about the a, b or c axis of the cubic unit cell. A recent piezoresponse force microscopy study of (110) oriented MAPbI_3_ films shows images of such ferroelastic domains at room temperature^53^. The domain spacing varied from 100 nm to 350 nm in different grains, but the domain walls were not resolved in that study. However, since the crystal structures within the domain walls are expected to differ from the structures within the domains themselves, their electronic properties will also differ. In perovskite oxides, conducting domain walls within insulating domains, and insulating domain walls within conducting domains, have been observed in different materials^54^. Thus the presence and density of domain walls can clearly have a direct impact on conductivity, and this possibility has also been theoretically explored in the hybrid perovskites^55^,^56^,^57^. There it has been suggested that the presence of charged ferroelectric domain walls with reduced bandgaps lead to enhanced charge separation and transport, and improved PV performance^55^. However, an important result of our study is the finding that the cubic-tetragonal transition for MAPbI_3_ is close to tricritical. It has been pointed out that the density of domain walls is expected to be significantly smaller for a tricritical phase transition, compared to a second-order phase transition, especially near the critical temperature^33^. Whether such a decreased domain wall density helps or hinders PV performance depends upon the electronic properties of the domain walls, which have yet to be characterized experimentally in the hybrid perovskites to the extent that domain walls in other ferroics have recently been characterized^54^. To do so will require characterization of the structural phase transitions (by x-ray diffraction or other techniques), the domain structures (by optical and force microscopies and scattering techniques), and the transport, dielectric and PV properties, preferably for the same set of samples. Furthermore, it may be possible to tune the critical behaviors and temperatures of the phase transitions, and the resulting domain structures, by techniques such as doping or applying strain through the substrate. This type of domain wall engineering is an active area of research in ferroic materials in general^54^ and may become important for hybrid perovskites as well. In this contribution we have shown that neutron and synchrotron x-ray scattering can provide both the sensitivity to the inorganic and organic components and the very high resolution necessary to characterize the structures and structural phase transitions of hybrid perovskites.

A second important aspect of the phase transitions is their possible impact on electron-phonon coupling, which is likely to be the dominant source of charge carrier scattering in the hybrid perovskites, but is still not fully understood. Measurements of scattering rates by terahertz spectroscopy of photoexcited carriers have provided evidence for acoustic phonon deformation potential scattering over the temperature range of photovoltaic operation for the tetragonal phase^58^. More recent work based upon photoluminescence linewidths has instead led to the conclusion that longitudinal optical (LO) phonons provide the dominant scattering mechanism over that temperature range, by analogy with earlier results for polar semiconductors^59^. Interestingly, recent work on doped SrTiO_3_ has shown that electron-LO phonon couplig dominates at high temperature, and acoustic phonon scattering at low temperature, but at intermediate temperatures transverse optical phonon scattering associated with the soft modes that drive the antiferrodistortive phase transition at T_c_ = 105 K plays an important role^60^. Our work has shown that the cubic-tetragonal transition in MAPbI_3_ is driven by an R-point soft mode, just as the cubic-tetragonal transition in SrTiO_3_, while the tetragonal-orthorhombic transition is primarily driven by a soft mode at the M-point in MAPbI_3_. Since it is likely that the phonons along the entire Brillouin zone M-R line in MAPbI_3_ are soft and have large vibrational amplitudes, it is possible that charge carrier scattering with these modes also contributes significantly to the electrical transport properties. These soft modes are transverse acoustic phonons in the high temperature phase, but fold over to the Brillouin zone center below the phase transition to become tranverse optical modes, as in SrTiO_3_. In addition, the soft PbI_6_ modes are coupled to the highly disordered MA polar cations, which may also impact the charge carriers in MAPbI_3_. Other transport properties, for example thermal conductivity, can also be affected by the presence of lines of soft modes in reciprocal space and the strong anharmonicity associated with such phonons.

Finally, we have also provided values for the average thermal expansion coefficients of the cubic and tetragonal phases of d_6_-MAPbI_3_ and h_6_-MAPbI_3_ (see captions to Fig. 6c and Supplementary Fig. 10a) for the temperature range where PV devices will operate. Since these devices will be exposed to significant temperature changes, it is important to try to minimize the mismatch between these values and those of the surrounding components of the device to obtain longer device lifetimes. These values will facilitate the design of more stable devices based upon this hybrid perovskite.

## Methods

### Sample preparation

All samples were prepared in a nitrogen glovebox. Phase purity of all samples was confirmed by X-ray powder diffraction.

### Starting Materials

The deuterated starting materials were obtained from Sigma-Aldrich. The methyl ammonium chloride was 98 atom % D, as was the DI gas. The methyl-d_3_ ammonium-h_3_ lead iodide was prepared using methyl-d_3_ amine-h_2_ that was 99 atom % D. The methyl ammonium-d_3_ iodide was prepared staring with methylamine gas that was first reacted with HI to yield methyl ammonium iodide. The H atoms attached to the nitrogen atoms were then exchanged with D by dissolving the salt in 10 ml D_2_O (99 atom % D), drying under vacuum, and then repeating two more times. The resulting CH_3_ND_3_I was estimated to be better than 98 atom % D on the ammonium group. Lead iodide of 99% purity was obtained from Acros Organics.

### CD_3_ND_3_PbI_3_

To prepare the fully deuterated methyl ammonium lead iodide, 1.0 g of methyl ammonium chloride (CD_3_ND_2_ DCl) was dissolved in 15 g of 16% DI in D_2_O (made by dissolving 5 g DI gas in 25 g D_2_O) and pumped to dryness to yield CD_3_ND_2_ DI. This material was added to 6 g of lead iodide and mixed in ~25 mL of DMF. Upon stirring, the slurry immediately dissolved to yield a pale yellow clear solution. The resulting solution was warmed then stirred overnight in a N_2_ glove box. The solution was evaporated to dryness under vacuum and the resulting black solid was washed with dichloromethane and n-propanol and then isolated by suction drying. The solid was then annealed in nitrogen in the glove box at 140 °C for 1 hr to remove residual solvent. This yielded 7.95 g of d_6_-MAPbI_3_ (97% yield). Thermogravimetric analysis showed that there was less than 0.1 wt% solvent released up to temperatures of 200 °C.

### Neutron Powder Diffraction

Samples were loaded into 8 mm diameter vanadium cans in a helium glovebox for analysis on the POWGEN diffractometer^61^ situated at the Spallation Neutron Source, Oak Ridge National Laboratory. The sample size was ~5.5 g for the d_6_-MAPbI_3_. To collect data over a sufficient range of d-spacing, two frames with center-wavelengths of 1.066 Å and 2.665 Å were collected, yielding a minimum d-spacing of 0.3 Å and a maximum of 9.2 Å. Two sample environments were used to cover the desired temperature range of 10–350 K. Data at temperatures between 10 K and 300 K data were collected using a sample autochanger equipped with a closed cycle refrigerator. For temperatures between 300 K and 350 K a top-loading Janis cryofurnace was used.

To minimize the effects of diffuse scattering the best quality data for the orthorhombic and tetragonal structures were collected at 10 K and 190 K, respectively. Additional datasets were collected at 130 K (orthorhombic) and 300 K (tetragonal). For the high temperature cubic phase a high quality dataset for refinement was collected at 350 K.

The occupancy of the lead atom had to be refined at T = 10 K as the values of the displacement parameters were so small that they refined to non-physical values. Lead is one element for which there is considerable natural geographic variability of the isotope abundances. Depending on the geological history of the source rock the concentrations of the different radiogenic lead isotopes, ^206^Pb, ^207^Pb and ^208^Pb in an ore can vary^62^. Since the different lead isotopes have different neutron scattering lengths, the average Pb scattering length for different samples can vary as well. Neutron diffraction measurements are only sensitive to the total site scattering length so this leads to some uncertainty in lead site occupancy without knowing the isotopic ratio of a sample. Consequently, the lead occupancy was refined for the d_6_-MAPbI_3_ at 10 K versus the nominal lead scattering length leading to a value of 1.011(3) Å. The lead occupancy was fixed at this value for subsequent refinements.

The analyses of the POWGEN data were carried out using the TOPAS refinement package^63^. CIF files for the refined structures have been deposited with the Cambridge Crystallographic Data Centre (CCDC 1509007-1509013). Diffuse scattering was visible from d_6_-MAPbI_3_ even down to 10 K. This became more pronounced with increasing temperature, creating problems during many of the refinements. The number of resolvable reflections also decreased with temperature so the fitted ranges were truncated to remove as much redundant background as possible. The detector layout of POWGEN when these data were collected (2014–15) yielded much better counting statistics at backscattering (i.e. low d-spacing or high Q), meaning that poor fitting of the high Q background could lead to poor structural parameters. At 10 K the oscillations could be handled satisfactorily with conventional background parameters but at higher temperatures (130 K and above) a sin(Qr)/Qr background function was used to reduce the number of background parameters needed to produce a reasonable fit. A single sin(Qr)/Qr function is only a gross approximation given that multiple correlation lengths exist within the structure, so additional measures were required at higher temperatures to extract meaningful ADPs from the MA cation and framework simultaneously.

The TLS (Translation, Libration, Screw) approach to restraining anisotropic displacement parameters^19^ is commonly used in macromolecular crystallography to reduce the number of refinable parameters whilst still obtaining information on thermal motions^64^. Its successful application in powder diffraction has been demonstrated in the literature^65^, but has seen little use despite being supported in Rietveld packages. A TOPAS input file containing the TLS restraints was modified such that the actual refined variables were those described by Rae^27^.

The change in basis of the tetragonal *I4/mcm* space group makes direct comparison between the *Pnma* and *I4/mcm* structures less straightforward. The long axis lies in the b direction in the case of *Pnma* and the c direction in the case of *I4/mcm*. To maintain consistency across both structures the iodine atom labels were switched from those used by Weller *et al.*^14^ for the tetragonal phase. The MA cation in the tetragonal structure was highly disordered with small occupancies for each site due to the four-fold site symmetry. To keep the structure physically reasonable, the number of refined parameters was reduced by describing the MA cation as a z-matrix rigid body with restraints used for refining bond lengths. A refinable torsion of the rigid methyl group with respect to the ammonium group was included as part of the z-matrix description. TLS restraints define a point about which the effects of the motions are centered. The starting points of the center-of-action of the MA TLS rigid bodies were assumed to be at the center-of-gravity of the MA cation, which is slightly closer to the heavier ammonium group. The D:H ratio was refined for the 10 K data and this value applied as a constraint in the other refinements of the fully deuterated sample. The use of a restrained z-matrix meant that bond distances and angles would have remained in line with expectations, where a free refinement would have been severely over-parameterized.

Despite the presence of the molecular MA cation, the perovskite framework could still be described within the well-defined tilt-systems previously described^6^,^7^,^9^. The distortions of the orthorhombic and tetragonal structures could be decomposed with respect to the parent cubic *Pm-3m* structure into symmetry-based distortion modes. The distortion-mode amplitudes relate to particular actions within the structure such as rotations, twists and displacements. The structure could be refined in TOPAS with the mode amplitudes replacing atomic coordinates as the refined parameters^66^. Taken in tandem with the molecular TLS description of the MA group, this resulted in a hybrid refinement reflecting the hybrid nature of the structure.

The phase behavior was studied by collecting data continuously using the 2.665 Å frame while cooling and heating the samples in the sample changer between 300–10 K at a nominal rate of 1.0 K/min and heating to 350 K at a nominal rate of 0.5 K/min in the cryofurnace. In order to prevent gaps in data due to accelerator dropouts a dynamic feedback between the accumulating proton charge and ramp-rate has been implemented at POWGEN such that near-constant counting statistics are maintained across the temperature range. In practice this means that the sample temperature is held steady if the beam drops out and will resume with a speed dependent on the rate at which the power is ramped up to operating conditions. Given the very low thermal conductivity of MAPbI_3_^67^ transition temperatures are likely to be affected by heating rate and some hysteresis was expected to a greater or lesser degree.

The orthorhombic-to-tetragonal transition was studied in detail by using a 2.665 Å center wavelength dataset where very slow ramping rate of 0.25 K/min was utilized between 156–176 K. The data were rebinned into datasets every 0.5 K with a nominal counting time of 2 minutes. In order to obtain useful structural information from noisy data with d_min_ of 1.1 Å, constraints and restraints had to be applied in such a way to maximize the information that could be extracted without over-constraining the refinement. A number of approaches were tried to study the behavior of the refinement with different constraint/restraint methodology. The approach settled on for the final results used combined ISODISTORT and TLS restraints. Einstein behavior of the displacement parameters was parameterized using results from the 10 K refinement. Lead and iodine were assigned initial isotropic displacement parameters equivalent to the 10 K refinement result. The initial TLS parameters from a refined of the 10 K dataset were utilized.

All 40 datasets were refined simultaneously in a two-phase surface refinement. Structural parameters for the tetragonal phase were taken from the 190 K structure refinement and fixed. Parameters refined individually for each dataset included the two scale factors (where two phases were observed to exist), the 3 strain mode amplitudes (GM_1_^+^, GM_2_^+^ and GM_3_^+^) and the displacement mode parameters R_4_^+^, R_5_^+^, M_3_^+^ and X_5_^+^. Decomposition of the 10 K *Pnma* structure versus the ideal *Pm-3m* structure using the Isotropy suite^34^ yielded almost zero amplitude for M_2_^+^, so it was fixed at zero for the series of refinements. The temperature dependences of the various distortion and strain modes are shown in Supplementary Fig. 12.

In order to reproduce the behavior observed in the raw data the phase fractions in the data from 169–175.5 K were parameterized using the Kolmogorov-Johnson-Mehl-Avrami rate equation:


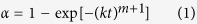


where k is the overall rate constant, t is time and (m+1) is the Avrami exponent^68^. The use of a constant ramp-rate meant time and temperature information could be extracted from a single dataset. The heating rate used to collect the data in this case was very low and the δT across the transition was fairly small, so the isothermal assumption in the Avrami rate equation was closely approximated.

A similar surface refinement was carried out using data sliced every 0.5 K from 300 K up to the tetragonal-cubic transition in order to extract the temperature-dependence of the R_4_^+^ mode amplitude, and therefore the order parameter.

### Synchrotron X-Ray Powder Diffraction

The X-ray powder diffraction measurements were performed on the bending magnet station at DND-CAT sector 5 of the Advanced Photon Source. The X-ray wavelength used was 0.40012(2) Å, selected to reduce X-ray absorption by the sample to an acceptable level. The wavelength was calibrated using a Si standard. The sample data were collected in flat plate geometry, with the Cu plate installed in an Advanced Research Systems cryostat and collected over a temperature range of 12 K to 350 K. Data were collected using a 1-D solid state Cyberstar detector in Bragg-Brentano geometry. Typical step sizes in two-theta were 0.0015 deg.

The X-ray data were analyzed by fitting the Bragg peaks with functions that were sums of Lorentzians and Gaussians, along with a linear background function. Peak locations, intensities and widths were the fitted parameters, and these values were used to evaluate the tetragonal strain and superlattice Bragg peak intensities versus temperature, and thus the temperature dependent order parameters, for the phase transitions.

### Neutron Pair Distribution Function Analysis

Neutron Pair Distribution Function (PDF) data was collected at the Nanoscale Ordered Materials Diffractometer^69^ at the Spallation Neutron Source, Oak Ridge National Laboratory. Two sample environments were used to cover the desired temperature range of 5–350 K. Data at temperatures between 5 K and 300 K data were collected using a cryostat equipped with He dilution refrigeration utilizing the same ~5.5 g d_6_-MAPbI_3_ sample in an 8 mm diameter vanadium can as in the POWGEN measurements. Two hour measurements were completed every 25 K, and additionally at 5 K, 160 K, and 190 K. For temperatures between 300 K and 350 K a top-loading sample changer with an Ar cryostream was utilized. For these measurements a powder sample was loaded into a 2 mm quartz capillary sealed with fiber glass wool and epoxy inside a He filled glovebox. Measurements were completed every 4 to 5 degrees for 30 minutes.

The NOMAD data reduction software^69^ was used to produce normalized total scattering patterns, S(Q), applying detector calibrations, normalizing by scattering from a vanadium rod (corrected for absorption and multiple scattering), and subtracting background scattering from the empty container and instrument. Inelastic incoherent effects resulting from hydrogen scattering in the samples were corrected prior to Fourier transformation via the method of nonlinear least-squares fitting to a pseudo-Voigt function. The details of the correction impacted the intensities, but not the position, of pair correlations below ~2.5 Å; intensities above this range were not affected. Pair distribution function patterns, G(r), were produced utilizing a Q maximum of 25 Å^−1^. G(r) simulations and least squares real-space modeling of the experimental G(r)s were completed in PDFgui^70^. A Ni dataset was fit between 1 and 50 Å to refine the instrument specific parameters, Q_damp_ = 0.021 Å−1 and Q_broad_ = 0.022 Å−1, and these were held fixed during refinement. Data were fit between 0.7 and 40 Å utilizing model parameters for a scale factor, lattice parameters and a parameter to account for correlated motion effects at low real-space ranges. Model fits were attempted with refinement of atomic positions, anisotropic atomic displacement parameters, and site occupancy of shared atom sites. Temperature-dependent differences in the collected PDF patterns were examined by producing contour plots for the series of data collected in the liquid helium orange cryostat (5 K through 300 K) and the NOMAD sample shifter equipped with an argon cryostream (296 K through 350 K).

### Simulations

Ab initio simulations based on density functional theory (DFT) were performed using VASP^71^ with plane-wave basis. The Generalized Gradient Approximation (GGA), as implemented by Perdew–Burke–Ernzerhof (PBE)^72^, was employed for the exchange-correlation functional. The corresponding Projector Augmented-Wave (PAW) pseudopotentials^73^ were used, with an energy cutoff of 900 eV. The lattice constants and atomic coordinates of the experimentally measured 10 K structure at POWGEN were relaxed to the local potential energy minimum, with and without the symmetry constraints. The electronic structure was calculated on a 7 × 5 × 7 Monkhorst-Pack grid, and the tolerance for electronic energy minimization was 10^−9^ eV/atom. The interatomic forces after relaxation were below 0.005 eV/Å, and the stresses were below 0.05 GPa. The optB86b-vdW functional^74^ for dispersion corrections was applied. The vibrational eigen-frequencies and modes were then calculated using the density functional perturbation theory (DFPT).

## Additional Information

**How to cite this article**: Whitfield, P. S. *et al.* Structures, Phase Transitions and Tricritical Behavior of the Hybrid Perovskite Methyl Ammonium Lead Iodide. *Sci. Rep.* **6**, 35685; doi: 10.1038/srep35685 (2016).

## Supplementary Material

Supplementary Information

## Figures and Tables

**Table 1:** Order parameters and distortion modes for the three crystal structures of MAPbI_3_.

Space Group Stability Range Pseudo-cubic Unit Cell Parameters	Order Parameters		Lattice Vectors	Glazer
	M_3_^+^ k = (½ ½ 0)	R_4_^+^ k = (½ ½ ½)		
*Pm-3m* (No. 221) T > 330 K a_c_ × a_c_ × a_c_	000	000	100 010 001	a^0^a^0^a^0^
*I4/mcm* (No. 140) 160 K < T < 330 K √2a_c_ × √2a_c_ × 2a_c_	000	q_4_00	110 1–10 002	a^0^a^0^c^-^
*Pnma* (No. 62) T < 160 K √2a_c_ × √2a_c_ × 2a_c_	0q_2_0	q_4_0q_6_ (q_2_ ≠ q_4_ = q_6_)	110 002 1–10	a^-^b^+^a^-^

R_4_^+^ (also referred to as the R_25_ mode) is active in the *Pm-3m* → *I4/mcm* transition and involves an out-of-phase rotation of magnitude q_4_ about the cubic *a* axis. Both M_3_^+^ and R_4_^+^ are active in the *I4/mcm* → *Pnma* transition: an in-phase rotation of magnitude q_2_ around the cubic *b* axis plus an out-of-phase rotation of magnitude q_6_ around the cubic *a* and *c* axes. The space groups, approximate temperature ranges of stability, and pseudocubic unit cell parameters are given in the first column; the distortion modes, k-vectors and order parameters in the second and third columns; the lattice vectors in the fourth column; and the Glazer notations for the three tilt structure in the last column.

**Table 2:** Isotope shifts and thermal hysteresis measurements for structural phase transitions of MAPbI_3_.

Sample	Structural Transition	Transition Temperature (K) (heating)	Transition Temperature (K) (cooling)	Hysteresis (K)
**CD**_**3**_**ND**_**3**_**PbI**_**3**_^a^	*Pm-3m* → *I4/mcm*	330	315	15
	*I4/mcm* → *Pnma*	172	163	9
**CD**_**3**_**ND**_**3**_**PbI**_**3**_^b^	*Pm-3m* → *I4/mcm*	332	332	0
	*I4/mcm* → *Pnma*	—	160–165	—
**CH**_**3**_**ND**_**3**_**PbI**_**3**_^a^	*Pm-3m* → *I4/mcm*	335	325	10
	*I4/mcm* → *Pnma*	169	162	7
**CH**_**3**_**NH**_**3**_**PbI**_**3**_^b^	*Pm-3m* → *I4/mcm*	338	338	0
	*I4/mcm* → *Pnma*	—	160–165	—

^a^Determined from neutron powder diffraction data. Heating and cooling rates were 1 K/min.

^b^Determined from synchrotron X-ray powder diffraction data. Temperatures were equilibrated for approximately 10 minutes after 1 K changes.
