# Corrigendum: Structures, Phase Transitions and Tricritical Behavior of the Hybrid Perovskite Methyl Ammonium Lead Iodide

**DOI:** 10.1038/srep42831

**Published:** 2017-02-21

**Authors:** P. S. Whitfield, N. Herron, W. E. Guise, K. Page, Y. Q. Cheng, I. Milas, M. K. Crawford

Scientific Reports 6: Article number: 35685; 10.1038/srep35685 published online: 10 21 2016; updated: 02 21 2017.

In this Article, there are errors in the figure legends:

In Figure 6c:

‘Lattice parameters determined from the synchrotron X-ray diffraction scans of the cubic 200 and tetragonal 220/004 Bragg peaks. Fits to the cubic lattice parameter (black solid line) and the average tetragonal lattice parameter (=2atet + ctet)/3 (blue solid line), were used to determine the linear coefficients of thermal expansion of 1.95 × 10^−4^ K^−1^ (cubic) and 2.66 × 10^−4^ K^−1^ (tetragonal). The region of cubic and tetragonal phase coexistence can be clearly seen’.

Should read:

‘Lattice parameters determined from the synchrotron X-ray diffraction scans of the cubic 200 and tetragonal 220/004 Bragg peaks. Fits to the cubic lattice parameter (black solid line) and the average tetragonal lattice parameter (2atet + ctet)/3 (blue solid line), were used to determine the linear coefficients of thermal expansion of 35(1) × 10^–6^ K^−1^ for the cubic phase (324–350 K) and 42.4(4) × 10^−6^ K^−1^ for the tetragonal phase (160–325 K). The region of cubic and tetragonal phase coexistence can be clearly seen’.

In Supplementary Figure 10a:

‘a) Lattice parameters as a function of temperature. The cubic lattice parameter (acub) and the average tetragonal lattice parameter (2atet + ctet)/3, were fit (blue and black solid lines, respectively) to extract the linear thermal expansion coefficients for the two phases, yielding 2.37(3) × 10^−4^ K^−1^ for the cubic phase and 2.65(1) × 10^−4^ K^−1^ for the tetragonal phase’

Should read:

‘a) Lattice parameters as a function of temperature. The cubic lattice parameter (acub) and the average tetragonal lattice parameter (2atet + ctet)/3, were fit (blue and black solid lines, respectively) to extract the linear thermal expansion coefficients for the two phases, yielding 37.6(5) × 10^–6^ K^−1^ for the cubic phase (328–350 K) and 42.2(3) × 10^–6^ K^−1^ for the tetragonal phase (160–325 K)’.

