# Supplementary material for: Structures, Phase Transitions and Tricritical Behavior of the Hybrid Perovskite Methyl Ammonium Lead Iodide
Source: Sci Rep. 2016 Oct 21;6:35685. doi: 10.1038/srep35685 (PMC5073364; doi:10.1038/srep35685)
Supplement: Supplementary Information [file srep35685-s1.pdf]

Structures, Phase Transitions and Tricritical Behavior of the Hybrid Perovskite Methyl Ammonium Lead Iodide

P.S. Whitfield[1*], N. Herron[2], W.E. Guise[3,4], K. Page[1], Y.Q. Cheng[1], I. Milas[3] and M.K. Crawford[3,5*]

[1] *Chemical and Engineering Materials Division, Neutron Sciences Directorate, Oak Ridge National Laboratory, Oak Ridge, TN 37831, U.S.A.*

[2] *DuPont Electronics and Communication Technologies, Wilmington, DE 19803, U.S.A.*

[3] *DuPont Central Research & Development, Wilmington, DE 19803, U.S.A.*

[4]*Advanced Photon Source, Argonne National Laboratory, 9700 S. Cass Avenue, Lemont, IL 60439, USA*

[5]*Department of Physics and Astronomy, University of Delaware, Newark, DE 19716, USA*

Contact information: whitfieldps@ornl.gov, norman.herron@dupont.com, mikeginil@aol.com, pagekl@ornl.gov, chengy@ornl.gov, ivanmilas@yahoo.com, mkcrawford987@gmail.com

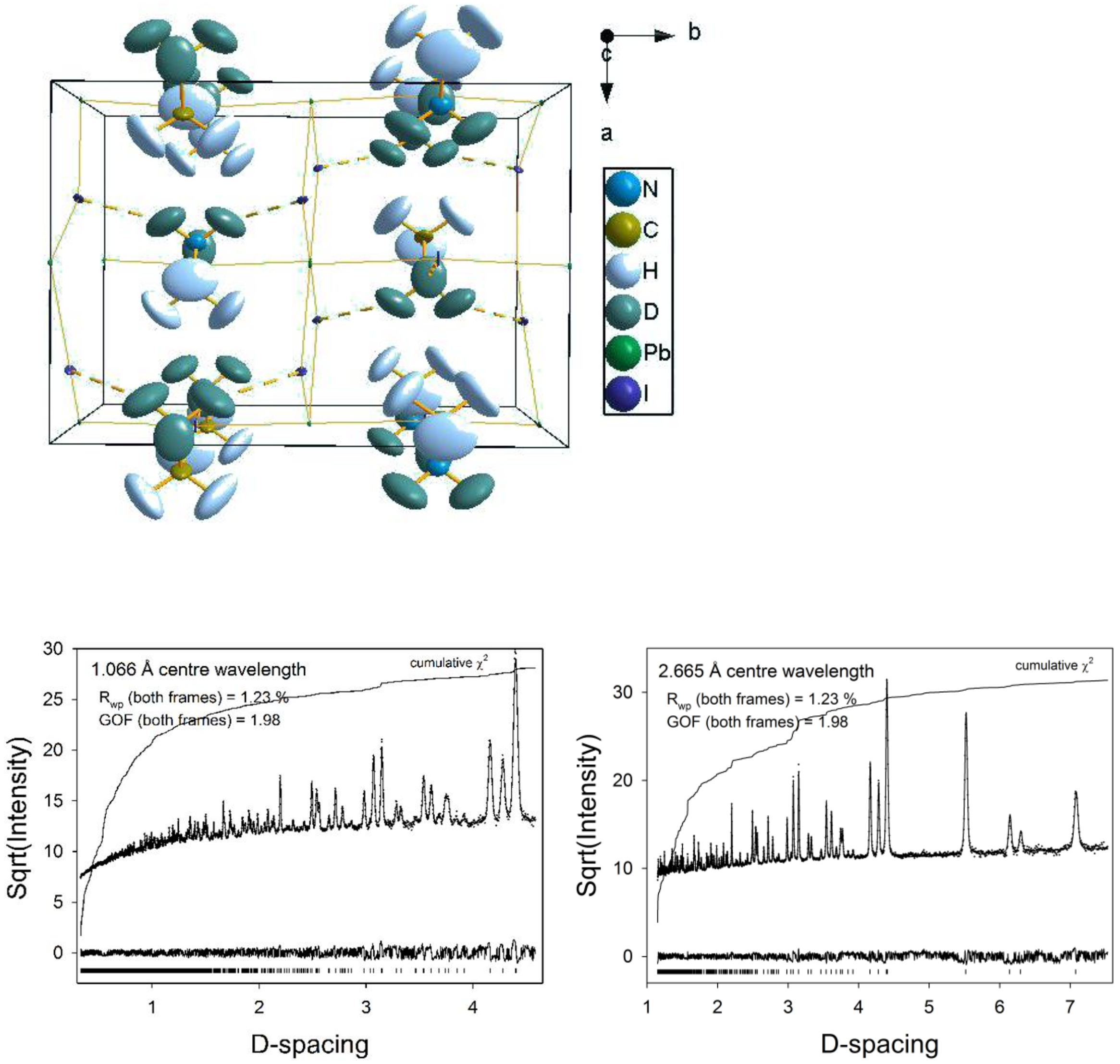

Supplementary Figure 1. Structure of d3-$CH_3ND_3PbI_3$ and fit to the 1.066 and 2.665 Å frame data at 10 K. Ellipsoids are drawn at 95% probability. The increased background from the incoherent scattering of the hydrogen can clearly be seen.

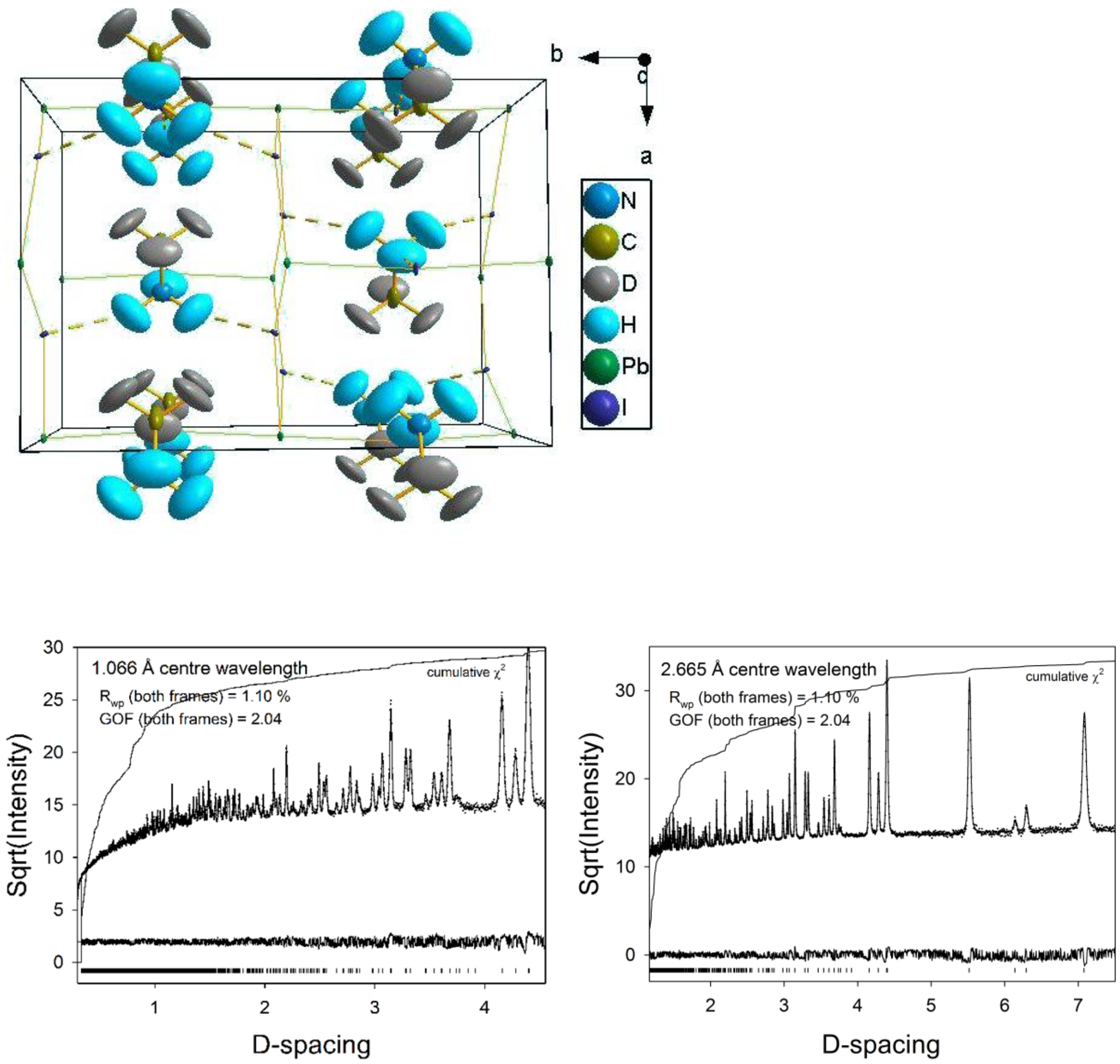

Supplementary Figure 2. Structure of d3-$CD_3NH_3PbI_3$ and fits to the 1.066 and 2.665 Å frame data at 10 K. Ellipsoids are drawn at 95% probability.

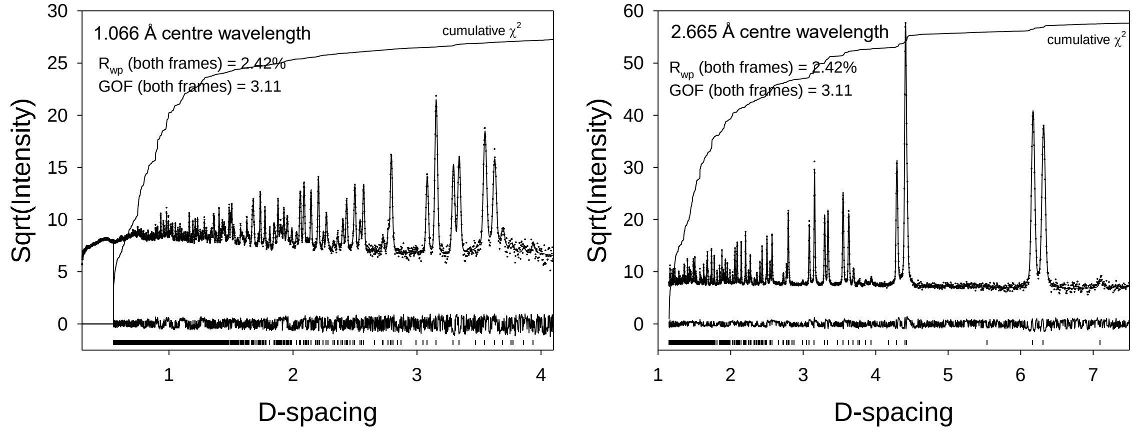

Supplementary Figure 3. Structure refinement for the neutron powder diffraction data for orthorhombic d_6-$CD_3ND_3PbI_3$ at 130K.

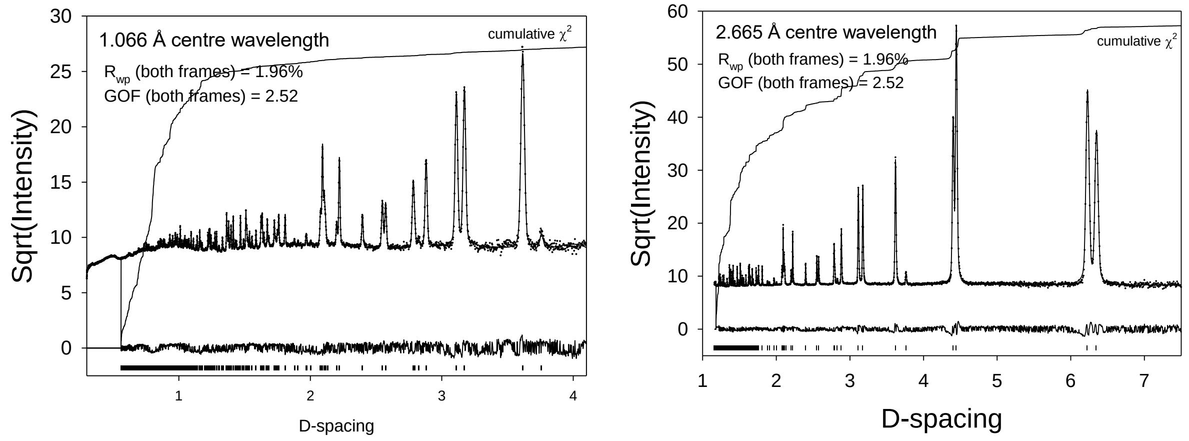

Supplementary Figure 4. Structure refinement for the neutron powder diffraction data for tetragonal d_6-$CD_3ND_3PbI_3$ at 190K.

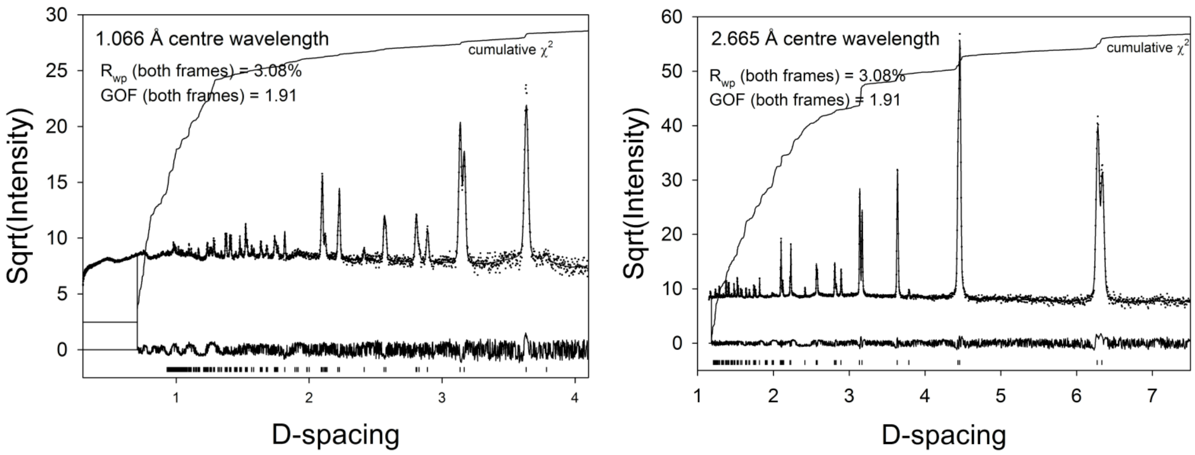

Supplementary Figure 5. Neutron powder diffraction data and Rietveld refinement for d$_6$-$CD_3ND_3PbI_3$ tetragonal phase at 300 K.

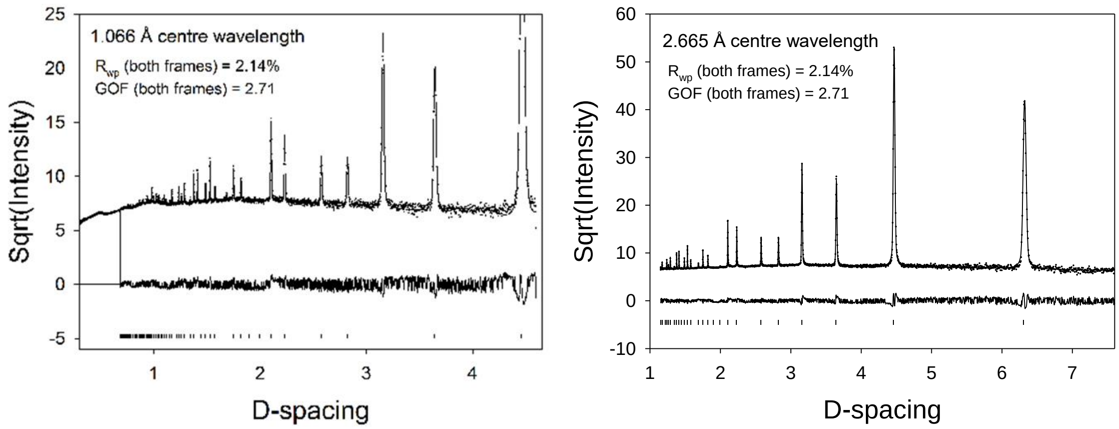

Supplementary Figure 6. Neutron powder diffraction data and Rietveld refinement for d$_6$-$CD_3ND_3PbI_3$ cubic phase at 350 K.

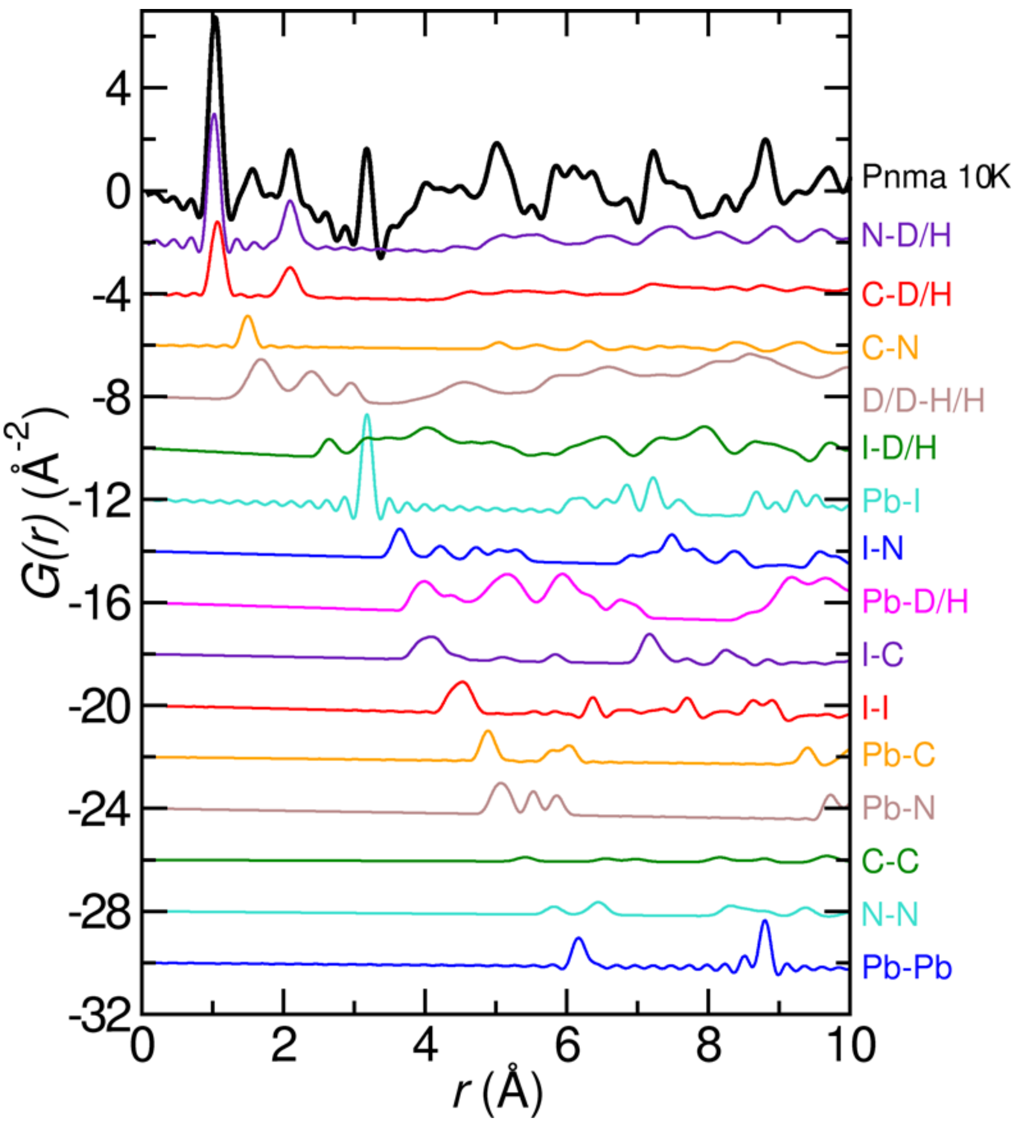

Supplementary Figure 7. PDF partials based on the 10 K average structure refinement.

a)

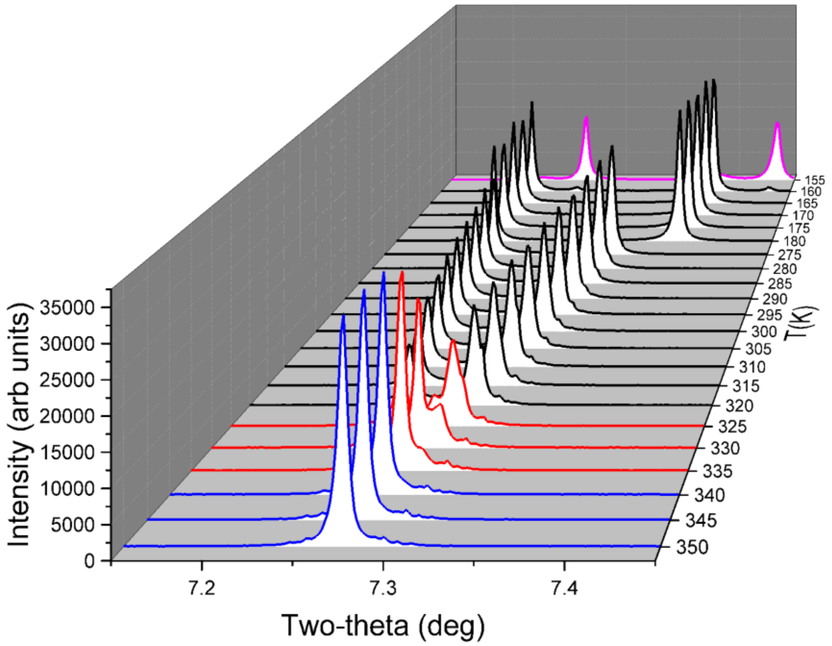
Intensity (arb units)
0
5000
10000
15000
20000
25000
30000
35000
7.2
7.3
7.4
Two-theta (deg)
T(K)
155
160
165
170
175
180
275
280
285
290
295
300
305
310
315
320
325
330
335
340
345
350

b)

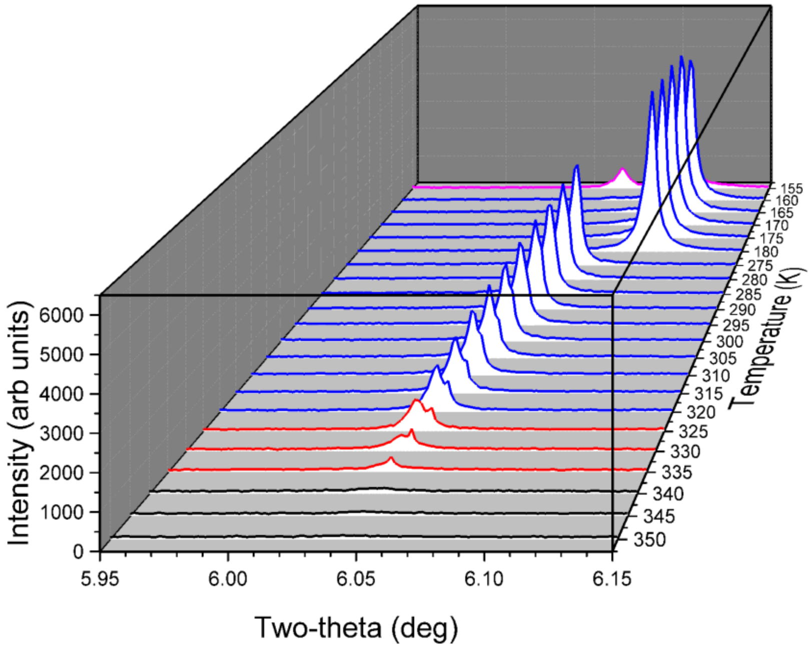
Intensity (arb units)
0
1000
2000
3000
4000
5000
6000
5.95
6.00
6.05
6.10
6.15
Two-theta (deg)
Temperature (K)
155
160
165
170
175
180
275
280
285
290
295
300
305
310
315
320
325
330
335
340
345
350

Supplementary Figure 8. Bragg peaks across the cubic-tetragonal phase transition for h_6-$MAPbI_3$. a) The cubic (200) and the tetragonal (220)/(004) Bragg peaks versus temperature. The curves shown in red have both tetragonal and cubic Bragg reflections present, demonstrating phase coexistence from roughly 320-335 K. Note that there is a break in the temperature scale between 275 K and 180 K. b) The (3 1 1) superlattice Bragg peak (referenced to the cubic unit cell doubled along all three axes) versus temperature.

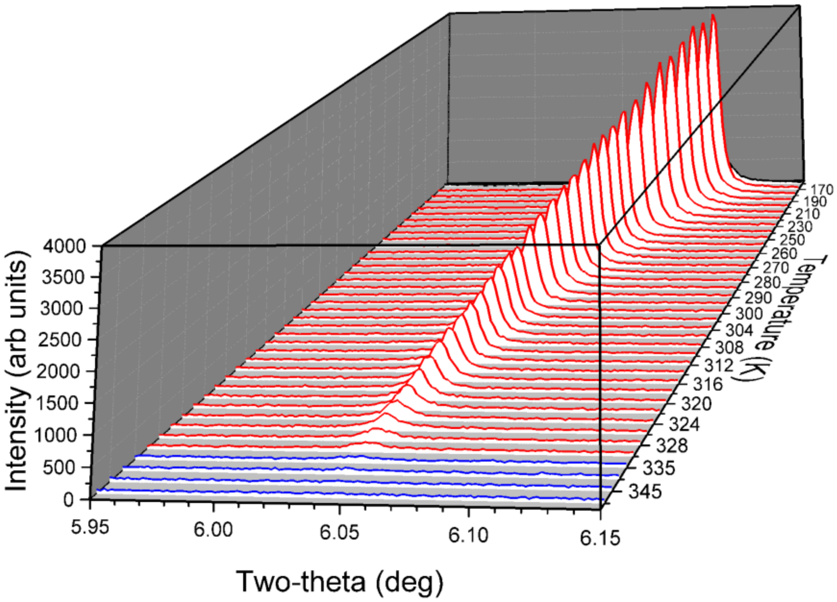

Supplemenary Figure 9. Superlattice Bragg peak across the cubic-tetragonal phase transition for d$_6$-MAPbI$_3$. The (311) superlattice Bragg peak (referenced to the cubic unit cell doubled along all three axes) versus temperature for fully deuterated MAPbI$_3$.

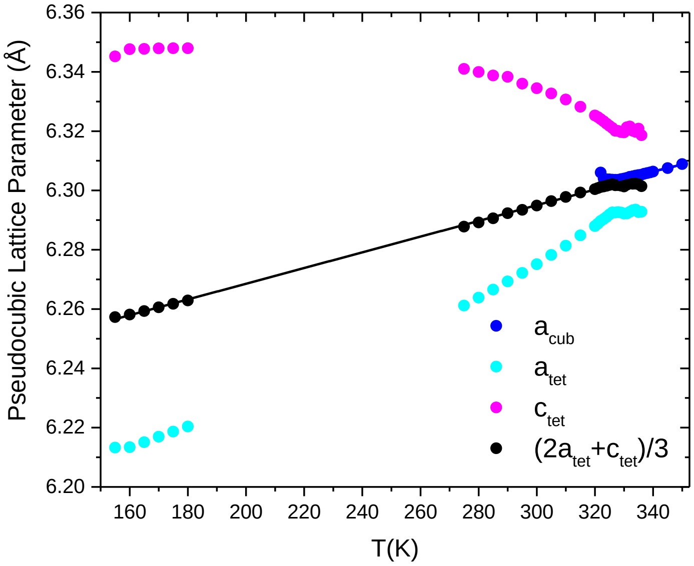

6.36
6.34
6.32
6.30
6.28
6.26
6.24
6.22
6.20
Pseudocubic Lattice Parameter (Å)
160
180
200
220
240
260
280
300
320
340
T(K)
a_{cub}
a_{tet}
c_{tet}
$(2a_{tet}+c_{tet})/3$

a)

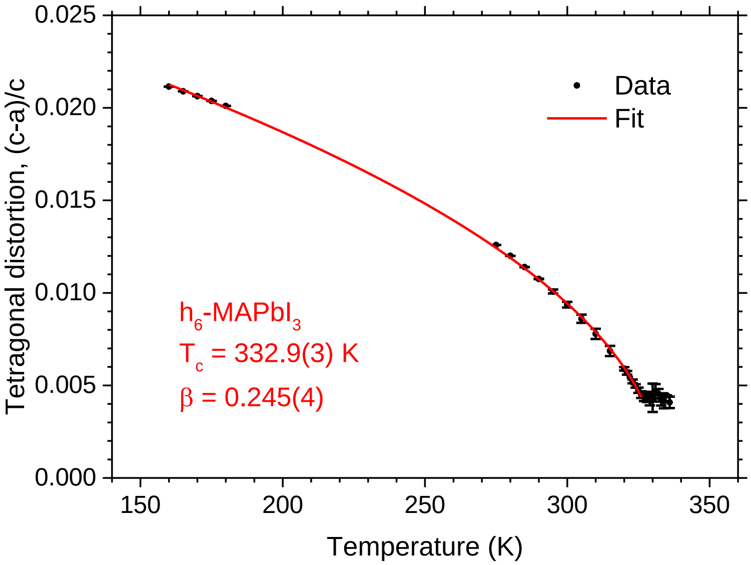

0.025
0.020
0.015
0.010
0.005
0.000
Tetragonal distortion, (c-a)/c
150
200
250
300
350
Temperature (K)
Data
Fit
h_6-MAPbI_3
T_c = 332.9(3) K
β = 0.245(4)

b)

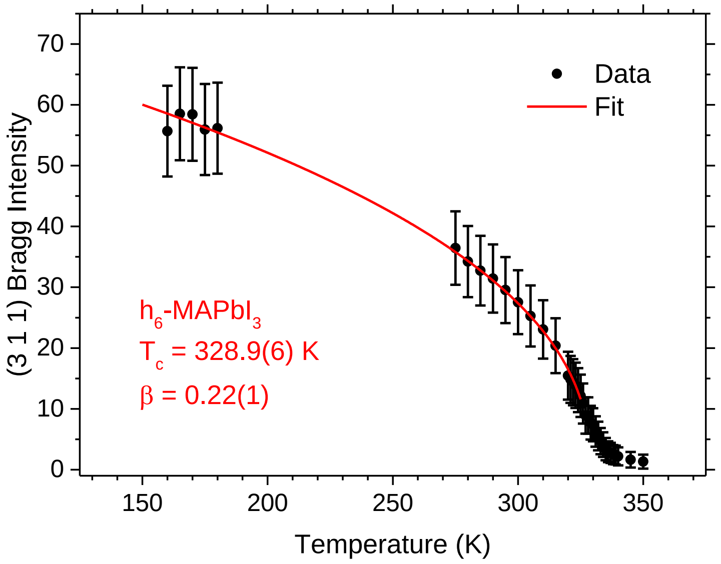

c)

Supplementary Figure 10. Synchrotron X-ray diffraction derived lattice parameters and order parameters across the cubic-tetragonal phase transition for h_6-$MAPbI_3$. a) Lattice parameters as a function of temperature. The cubic lattice parameter (a_{cub}) and the average tetragonal lattice parameter ($(2a_{tet} + c_{tet})/3$), were fit (blue and black solid lines, respectively) to extract the linear thermal expansion coefficients for the two phases, yielding 2.37(3) x 10^{-4} K^{-1} for the cubic phase and 2.65(1) x 10^{-4} K^{-1} for the tetragonal phase. b) Tetragonal distortion as a function of temperature. The red curve is a fit of the data to a power law, $(c-a)/a \sim (T-T_c)^{2\beta}$. Error bars are multiplied by 3 for visibility. c) Intensity of the (311) superlattice Bragg reflection (referenced to the cubic cell doubled along all three axes) that appears in the tetragonal structure. The data (black) is fit to a power law, $I_{311} \sim \text{constant} \times (T-T_c)^{2\beta}$, shown in red.

a)

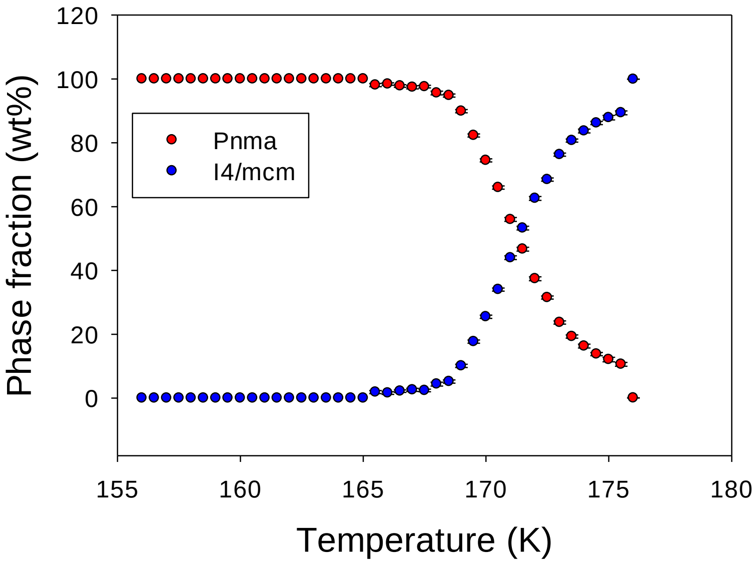
Phase fraction (wt%)
Temperature (K)
Pnma
I4/mcm
120
100
80
60
40
20
0
155
160
165
170
175
180

b)

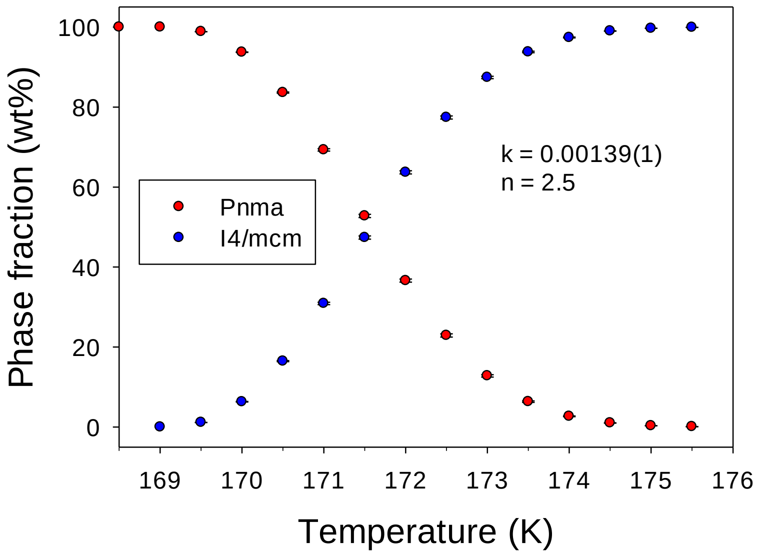
Phase fraction (wt%)
Temperature (K)
Pnma
I4/mcm
k = 0.00139(1)
n = 2.5
100
80
60
40
20
0
169
170
171
172
173
174
175
176

Supplementary Figure 11. Phase fractions of tetragonal and orthorhombic phases. a) Plot of the freely-refined orthorhombic and tetragonal phase fractions across the tetragonal-orthorhombic phase transition of d_6-$MAPbI_3$ based upon neutron diffraction data. b) Phase fractions across the orthorhombic-to-tetragonal transition when parameterized using the Avrami rate equation, as described in the Methods section.

a)

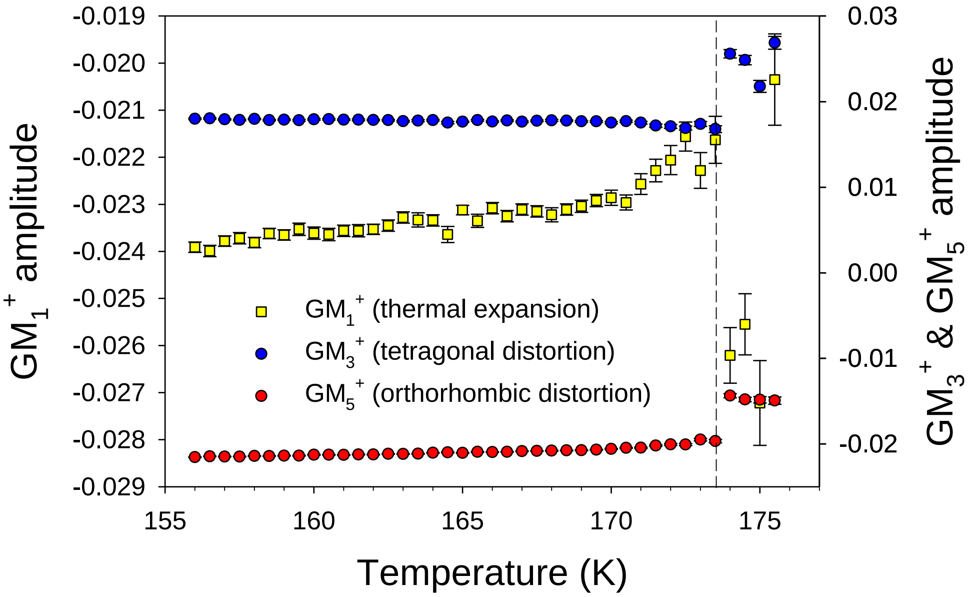

-0.019
-0.020
-0.021
-0.022
-0.023
-0.024
-0.025
-0.026
-0.027
-0.028
-0.029
GM1+ amplitude
0.03
0.02
0.01
0.00
-0.01
-0.02
GM3+ & GM5+ amplitude
155
160
165
170
175
Temperature (K)
GM1+ (thermal expansion)
GM3+ (tetragonal distortion)
GM5+ (orthorhombic distortion)

b)

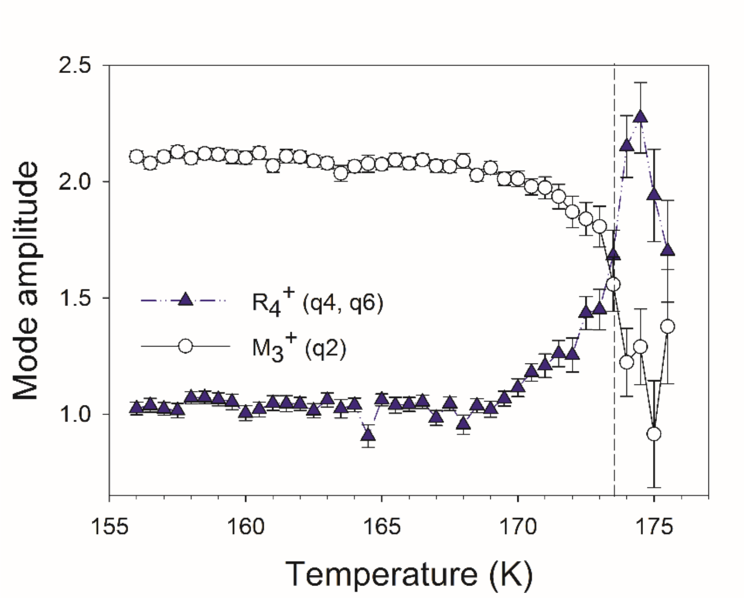

2.5
2.0
1.5
1.0
Mode amplitude
155
160
165
170
175
Temperature (K)
R4+ (q4, q6)
M3+ (q2)

c)

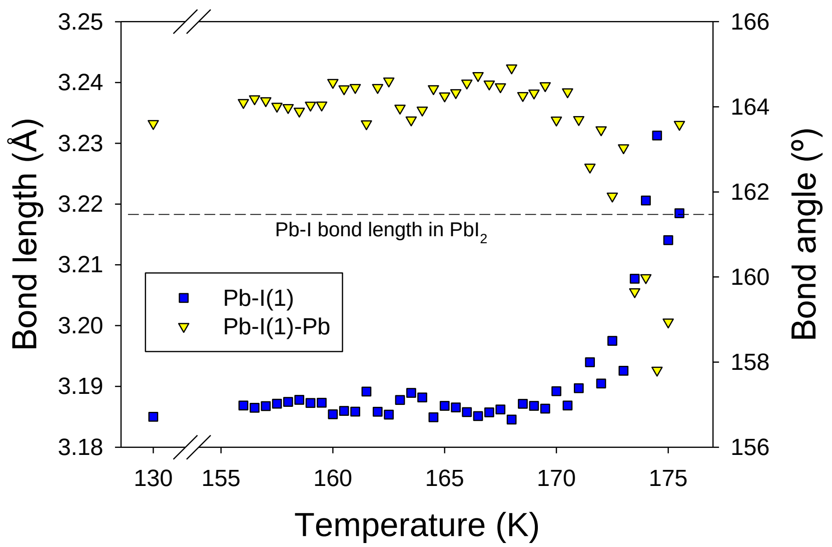

Supplementary Figure 12. Distortion and strain modes for $MAPbI_3$ on heating through the orthorhombic-tetragonal phase transition. a) Plot of the refined strain mode amplitudes versus the 350 K cubic lattice parameters across the orthorhombic to tetragonal phase transition from the Avrami-parameterized refinement. b) Plot of the refined M_3^+ and R_4^+ distortion mode amplitudes versus the parent cubic structure across the orthorhombic to tetragonal phase transition. c) Plot of the refined Pb-I(1) bond length and Pb-I(1)-Pb bond angle approaching the orthorhombic to tetragonal phase transition. The values from the refinement for the 130 K data are included for comparison. Also shown is the Pb-I bond length from trigonal PbI_2 at room temperature.

Pnma; a = 8.81155(6), b = 12.58714(9), c = 8.55975(6) Å , volume = 949.38(1) $Å^3$

R_{wp} = 2.14%, GOF = 2.71

Atom	Wyk	x	y	z	occ	u11	u22	u33	u12	u13	u23
Pb	4b	½	0	0	1.011(3)	12(3)	9(3)	5(3)	0	0	6(3)
I(1)	4c	0.0155(2)	¼	-0.5537(2)	1	77(7)	4(5)	29(6)	0	0(5)	0
I(2)	8d	0.3128(1)	0.01664(8)	-0.3164(1)	1	25(4)	24(4)	25(4)	-7(3)	-14(3)	4(3)
C	4c	0.0832(2)	¼	-0.0662(2)	1	37(6)	77(6)	117(7)	0	39(5)	0
D1	4c	0.0526(2)	¼	-0.1888(2)	0.937(2)	208(10)	400(13)	67(8)	0	48(7)	0
H1	4c				0.063(2)						
D2	8d	0.1455(2)	0.1794(1)	-0.0384(2)	0.937(2)	201(7)	213(7)	346(9)	150(5)	87(6)	75(6)
H2	8d				0.063(2)						
N	4c	-0.0605(1)	¼	0.0276(1)	1	63(4)	79(5)	50(4)	0	7(3)	0
D3	8d	-0.1246(2)	0.3157(1)	0.0016(2)	0.937(2)	210(7)	233(8)	284(7)	156(6)	32(7)	62(7)
H3	8d				0.063(2)						
D4	4c	-0.0385(3)	¼	0.1448(2)	0.937(2)	313(13)	306(12)	27(7)	0	-6(7)	0
H4	4c				0.063(2)						

Supplementary Table 1. Structural parameters for the orthorhombic phase of d_6-$CD_3ND_3PbI_3$ from the refinement of the 10 K data. Anisotropic displacement parameter values should be multiplied by 10^{-4}.

Lattice parameters	Experiment	Calculation (error)	
		Pnma	*P1*
a (Å)	8.81155	8.850 (0.4%)	8.859 (0.5%)
b (Å)	12.58714	12.688 (0.8%)	12.691 (0.8%)
c (Å)	8.55975	8.564 (0.05%)	8.568 (0.1%)
α (°)	90	90	90
β (°)	90	90	90
γ (°)	90	90	90
Volume ($Å^3$)	949.38	961.63 (1.3%)	963.21 (1.5%)

Supplementary Table 2. Comparison of the refined lattice parameters with the *ab-initio* optimized structure in both *Pnma* and unconstrained *P1*using VASP. The % error in the optimized values are given in parentheses after the value.

	$CD_3ND_3PbI_3$ (d_6)	$CH_3ND_3PbI_3$ (d_3-ND_3)	$CD_3NH_3PbI_3$ (d_3-CH_3)
**N-'H' (Å)**	2x 1.026(2); 1.022(2)	2x 1.018(2); 1.022(3)	2x 1.036(3); 1.052(5)
**'H'-I (Å)**	2x 2.696(2); 2.624(2)	2x 2.709(3); 2.628(3)	2x 2.683(3); 2.592(5)
**N-I (Å)**	2x 3.625(1); 3.646(2)	2x 3.628(2); 3.650(2)	2x 3.624(1); 3.644(2)
**N-'H'-I (degrees)**	2x 150.6(1); 179.5(1)	2x 150.3(2); 179.0(2)	2x 151.0(2); 178.3(3)
**N-C (Å)**	1.499(2)	1.470(3)	1.479(3)
**C-'H' (Å)**	2x 1.072(2); 1.083(2)	2x 1.084(4); 1.093(6)	2x 1.078(2); 1.089(3)
**I(1)-Pb-I(2) (degrees)**	88.22(2)	87.99(3)	85.31(2)
**Pb-I(2)-Pb (degrees)**	150.08(4)	150.30(5)	150.01(4)
**Pb-I(1)-Pb (degrees)**	162.675	162.636	162.553
**Pb-I(1)**	3.1831(2)	3.1831(3)	3.1830(2)
**Pb-I(2)**	3.180(1)	3.180(1)	3.177(1)

Supplementary Table 3. Comparison of selected bond lengths and angles from the fully and partially deuterated samples at 10 K.

I4/mcm; a = 8.79948(6), c = 12.6882(1) Å , volume = 982.46(2) Å^3; R_{wp} = 1.96%, GOF = 2.52

Atom	Wyk	x	y	z	occ	u11	u22	u33	u12	u13	u23
**Pb**	4c	0	0	0	1.011	232(6)	232(6)	229(9)	0	0	0
**I(1)**	4a	0	0	1/4	1	532(10)	532(10)	127(13)	0	0)	0
**I(2)**	8h	0.2013(1)	0.2987(1)	0	1	367(8)	367(8)	566(12)	-209(8)	0	0
**C1**	16l	0.4734(3)	0.0266(3)	0.2680(4)	0.125	851(75)	809(311)	546(88)	-254(100)	149(133)	508(60)
**N1**					0.125						
**D1**	32m	0.4496(6)	0.1118(5)	0.2217(6)	0.117	2158(162)	741(98)	1666(208)	-84(152)	-193(284)	-1056(111)
**H1**					0.008						
**D2**	32m	0.3799(5)	-0.0271(4)	0.2861(6)	0.117	503(70)	2317(244)	1323(160)	-54(218)	464(96)	534(79)
**H2**					0.008						
**D3**	32m	0.5212(6)	0.0645(8)	0.3323(5)	0.117	1925(179)	1658(156)	691(84)	-872(92)	135(206)	386(144)
**H3**					0.008						
**N2**	16l	0.5795(3)	-0.0795(3)	0.2132(4)	0.125	359(60)	246(54)	321(42	-51(4)	166(45)	57(33)
**C**					0.125						
**D4**	32m	0.6775(6)	-0.0298(5)	0.2030(8)	0.117	583(68)	1005(130)	970(95)	33(152)	511(69)	-257(52)
**H4**					0.008						
**D5**	32m	0.5933(9)	-0.1708(6)	0.2558(7)	0.117	580(126)	356(61)	1291(116)	307(78)	-44(176)	157(51)
**H5**					0.008						
**D6**	32m	0.5372(8)	- 0.1075(10)	0.1448(5)	0.117	1301(127)	929 (120)	450(53)	-477(65)	9(89)	131(110)
**H6**					0.008						

Supplementary Table 4. Structural parameters for the 8-fold disordered tetragonal phase from the refinement of 190 K data of d_6-$CD_3ND_3PbI_3$. Anisotropic displacement parameter values should be multiplied by 10^{-4}

Pm3m; a = 6.30649(3) Å, volume = 250.821(3) Å^3

R_{wp} = 2.84%, GOF = 1.97

Atom	Wyk	x	y	z	occ	u11	u22	u33	u12	u13	u23
**Pb**	1a	0	0	0	1.011	444(7)	444(7)	444(7)	0	0	0
**I**	3d	0.5	0	0	1	247(17)	1755(18)	1755(18)	0	0	0
**C**	6f	0.382(2)	½	½	1/6	1035(71)	1031(64)	1031(64)	0	0	0
**N**	6f				1/6						
**D**	48n	0.36(3)	0.549(5)	0.34(3)	0.1171	2410(1183)	2171(731)	2036(647)	272(214)	-1358(256)	-323(183)
**H**	48n				0.0079						

Supplementary Table 5. Structural parameters for the cubic phase from the refinement of 350 K data of d_6-$CD_3ND_3PbI_3$.

Anisotropic displacement parameter values should be multiplied by 10^{-4}.

	**10 K**	**130 K**	**190 K**	**300 K**	**350 K**
	Pnma		***I4/mcm***		***Pm-3m***
**Pb – I(1) (Å)**	3.1831(2)	3.1850(5)	3.169(1)	3.163(3)	3.153
**Pb – I(2) (Å)**	3.180(1)	3.175(2)	3.172	3.165	-
**Pb – I(1) – Pb (degrees)**	162.675	163.607	158.14(4)	164.7(1)	180
**Pb – I(2) – Pb (degrees)**	150.08(4)	151.53(7)	180	180	-
**I(1) – Pb – I(2) (degrees)**	88.22(2)	88.47(5)	90	90	90

Supplementary Table 6. Bond lengths and angles for the PbI_6 framework at various temperatures.

Detailed Symmetry-mode Descriptions

In addition to the R_4^+ and M_3^+ described in Table 1 and Figure 4 the *Pnma* structure can exhibit three other modes known as R_5^+, X_5^+ and M_2^+ [10]. Decomposition of the refined 10 K orthorhombic structure with ISODISTORT [34] showed the most active displacement modes in the orthorhombic structure to be R_4^+ and M_3^+, the pure PbI_6 octahedra tilt modes.

R_4^+ is the out-of-phase rotation of the octahedra around the cubic c-axis, located at the R point (½ ½ ½) at the boundary of the cubic Brillouin zone (BZ), giving the *I4/mcm* tetragonal structure, or in Glazer notation $a^0a^0c^-$. The pseudocubic lattice parameters in the *I4/mcm* structure are equal to $\sqrt{2}a_c \times \sqrt{2}a_c \times 2a_c$, where a_c is the cubic lattice parameter. Mode M_3^+ corresponds to the in-phase rotation of the octahedra around the cubic *c*-axis (Glazer $a^0a^0c^+$), located at the M point (½ ½ 0) at the boundary of the BZ, affecting the I(2) atoms only. The lattice parameters in the resulting *P4/mbm* tetragonal structure are $\sqrt{2}a_c \times \sqrt{2}a_c \times a_c$, where a_c is the cubic lattice parameter.

Modes R_5^+, X_5^+ and M_2^+ are not allowed in the tetragonal structure and only appear at the tetragonal-to-orthorhombic transition [10]. Mode R_5^+, located at the Brillouin Zone R-point, involves rotations of the octahedra around an axis intermediate to the *a* and *b* axes, causing the I(1) atoms to move in the x-y plane, and the I(2) and I(3) atoms to move parallel to the z direction. Finally, mode X_5^+, located at the X point on the BZ boundary, involves a translation of the I(1) atoms in the x-y plane, but no motion of the I(2) atoms, distorting the octahedra. The remaining but inactive M_2^+ mode would have the effect of moving the I(2) atoms closer to one of the Pb atoms, leading to two Pb-I(2) distances.

In Supplementary Information Fig. 12 we show the behavior of the main distortion and strain modes versus temperature on heating. Thermal hysteresis (Table 2) meant the transition

temperature shifted relative to that on cooling. The strain-mode amplitudes exhibited a transition occurring at about 173 K with a possible small kink at 170 K in the thermal expansion (GM_1^+). The M_3^+ mode showed a single discontinuity at 173 K, while the R_4^+ tilt and R_5^+ distortion (not shown) exhibited possible additional discontinuities at 170 K in addition to the transition at 173 K. It is interesting to note that the R_5^+ displacement amplitude dropped close to zero at 170 K. The plots of the Pb-I(1)-Pb bond angle and Pb-I(1) bond length in Supplementary Information Fig. 12 show the octahedra distorted rapidly on heating above 170 K, a consequence of the change in octahedral tilting

Landau Theory

The 2-4-6 Landau free energy *F(Q)* is given by [33,37]

$$F(Q) = \frac{1}{2}A(T - T_c)Q^2 + \frac{1}{4}BQ^4 + \frac{1}{6}CQ^6 \qquad (1)$$

where A, B and C are generally independent of temperature and Q is the order parameter. For the case of the cubic-tetragonal transition Q is the rotation angle of the PbI_6 octahedra. The value and sign of B determine whether the phase transition is continuous (B > 0), first-order (B < 0), or tricritical (B = 0). In the latter case, the minimum and stability conditions [37]

$$\frac{\partial F}{\partial Q} = Q[A(T - T_c) + BQ^2 + CQ^4] = 0 \qquad (2)$$

$$\frac{\partial^2 F}{\partial^2 Q} = A + 3BQ^2 + 5CQ^4 \geq 0 \qquad (3)$$

lead to

$$Q = \left[\frac{A}{C}(T_c - T)\right]^{\frac{1}{4}} \qquad (4)$$

Thus the order parameter should scale as temperature raised to the ¼ power for a tricritical phase transition [33,37].

In the first-order case with $B < 0$ and $C > 0$, there will be a temperature region of coexistence of the cubic and tetragonal phases given by [37]

$$\Delta T = T_2 - T_c = \frac{B^2}{4AC} \qquad (5)$$

where T_2 is the temperature at which the tetragonal phase first appears and T_c is the temperature at which the cubic phase vanishes and the sample is fully tetragonal. Obviously the temperature range of phase coexistence vanishes at the tricritical point, where $B = 0$.
